# Protective Role of *Morus alba* Extract Against *Vibrio cholerae*: Impacts on Growth, Biochemical and Enzymatic Responses, Haematoimmunology, and Tissue Histopathology in *Dormitator latifrons*

**DOI:** 10.3390/microorganisms13122784

**Published:** 2025-12-07

**Authors:** Yuniel Méndez-Martínez, Cesar Varas-Macias, Liceth Zambrano-Mamonte, Lizly Rengifo-Olvera, Saul Buri-Miño, William Gavilanes-Armijos, Paulette Hernandez-Marin, Veronica Segovia-Montesdeoca, Hector Cedeño-Blacio

**Affiliations:** Experimental Aquaculture Laboratory, Facultad de Ciencias Pecuarias y Biológicas, Universidad Técnica Estatal de Quevedo (UTEQ), Av. Quito, Km. 1 ½ vía Quevedo-Santo Domingo de los Tsáchilas, Quevedo 120150, Ecuador; cvarasm@uteq.edu.ec (C.V.-M.); liceth.zambrano2015@uteq.edu.ec (L.Z.-M.); lrengifoo@uteq.edu.ec (L.R.-O.); sburim@uteq.edu.ec (S.B.-M.); wgavilanesa@uteq.edu.ec (W.G.-A.); phernandezm@uteq.edu.ec (P.H.-M.); vsegoviam@uteq.edu.ec (V.S.-M.); hcedenob@uteq.edu.ec (H.C.-B.)

**Keywords:** antibacterial activity, antioxidant defense, bacterial challenge, disease resistance, mucosal immunity, phytobiotic supplementation, virulence gene expression

## Abstract

The use of phytobiotics in aquafeeds is a promising strategy to enhance performance and resilience to disease. This study evaluated the protective role of *Morus alba* (MA) extract against *Vibrio cholerae*, integrating in vivo responses in *Dormitator latifrons* (growth, biochemical and enzymatic responses, haemato-immunology and tissue histopathology) with in vitro assessment of *V. cholerae* growth, virulence-associated gene expression and cellular morphology. *D. latifrons* juveniles were fed five diets (0, 5, 10, 15 and 20 g/kg feed; three tanks per treatment, 15 fish per tank) for eight weeks, followed by a 7-day challenge with *V. cholerae*. MA increased growth and feed utilisation (*p* < 0.05); the 20 g/kg group reached 27.57 g final weight with a feed conversion ratio of 1.24, and whole-body protein and lipid contents rose at higher doses. MA modulated plasma biochemistry and key digestive (amylase, lipase), metabolic (ALT, AST) and antioxidant (SOD, CAT, GPx) enzymes, and improved haematological profiles. Histology of the intestine, liver and spleen showed preserved architecture and reinforced mucosal features in supplemented fish, particularly at 15–20 g/kg. Post-challenge, supplemented groups exhibited higher survival/relative protection than controls, alongside lower transaminases and stronger antioxidant responses. In vitro, MA extract inhibited *V. cholerae* growth, attenuated virulence-associated gene (*toxR, ompU*) expression and induced marked morphological damage in planktonic cells. Multivariate analyses (Z-score heatmaps and PCA) linked immune–enzymatic improvements with growth and protection. Overall, 15–20 g/kg MA optimised immunophysiological status and disease resistance, supporting MA as a functional feed additive for sustainable aquaculture of *D. latifrons*.

## 1. Introduction

Aquaculture has become a fundamental component for ensuring global food security, contributing significantly to the production of aquatic foods [1]. According to projections by the Food and Agriculture Organization of the United Nations (FAO), by 2032 global production of aquatic animals is expected to reach 205 million tonnes, reflecting an average annual growth rate of 1.6% and a projected per capita consumption of 21.3 kg [2].

The success of this industry largely depends on the appropriate selection of species with aquaculture potential, highlighting the importance of native species that have made significant contributions to strengthening and sustaining the sector [3,4]. *Dormitator latifrons*, a species native to estuarine environments of the eastern Pacific, from Palos Verdes in southern California, USA, to Peru, can reach a weight of up to 1.2 kg, according to FishBase [5]. This species has attracted interest due to its potential for regional aquaculture owing to its hardiness, acceptance of formulated feeds, and commercial and organoleptic value [6,7,8]. However, in certain Latin American countries, such as Ecuador, its culture faces significant challenges associated with bacterial diseases, particularly those caused by *Vibrio cholerae*, an opportunistic pathogen reported as a causal agent of outbreaks with high mortality rates [9,10,11].

Infections caused by *V. cholerae* in fish can lead to dermal lesions, haemorrhages, necrosis in internal organs and considerable mortalities, in addition to representing a zoonotic risk for consumers [12,13,14]. This pathogenic bacterium is among the emerging species of sanitary and productive relevance [10,15,16]. As a consequence, indiscriminate use of antibiotics has occurred in aquaculture, generating microbial resistance and adverse effects on the environment and human health [17,18].

In parallel, a growing body of evidence shows that plant-derived and other natural compounds can directly target *V. cholerae* through multiple antimicrobial and antivirulence mechanisms. Extracts from edible and medicinal plants rich in polyphenols and terpenoids disrupt *V. cholerae* membranes, increasing permeability, inducing leakage of intracellular contents and ultimately causing cell death [19,20,21]. Green tea epigallocatechin gallate and polyphenolic fractions from kombucha exert similar effects, damaging the outer membrane, generating oxidative stress and compromising energy metabolism in *V. cholerae* [20,22]. Essential oils and their major components, such as carvacrol and eugenol, inhibit growth, attenuate virulence factor production and reduce biofilm formation, partly by interfering with quorum-sensing networks and the expression of toxin- and adhesion-related genes [23,24,25].

Flavonoids including quercetin, naringenin, baicalein and fisetin have been shown to impair biofilm architecture and modulate intracellular signalling (for example, c-di-GMP) in multidrug-resistant *V. cholerae* isolates, reinforcing their potential as antivirulence agents rather than simple bactericidal molecules [26,27]. Moreover, polyphenols from traditional antidiarrhoeal plants can neutralise cholera toxin binding to GM1 receptors, providing an additional level of protection at the host–pathogen interface [28]. Within this framework, *Morus alba* (MA) extracts are particularly relevant because they combine antioxidant and antimicrobial properties and have demonstrated bactericidal activity against *V. cholerae*, supporting their exploration as functional ingredients to mitigate cholera-associated infections [29].

In this context, the search for sustainable strategies to improve growth, immunocompetence, and disease resistance in aquatic species has led to intensive exploration of alternatives, such as functional plant-derived compounds used as feed additives with bioactive properties [17,30,31]. Among phytochemical-rich plants, mulberry (*Morus* spp.), particularly *Morus alba* MA, has demonstrated a notable capacity to enhance digestive, immunological, and antioxidant physiology in fish and other animals [32,33,34]. Biologically, MA represents an unusually rich source of antimicrobial phytochemicals with documented activity against *V. cholerae* and other enteric pathogens. The leaves, fruits and root bark concentrate high levels of phenolic acids (e.g., chlorogenic, caffeic and gallic acids), flavonoids (rutin, quercetin, kaempferol), anthocyanins and polysaccharides, together with more specialised prenylated flavonoids and arylbenzofurans such as kuwanon C, kuwanon T, morusin and moracins [35,36,37].

These molecules combine strong antioxidant capacity with direct antibacterial effects, including disruption of bacterial membrane integrity, increased permeability, leakage of intracellular contents and interference with efflux pumps, as shown for prenylated phenolics from MA root bark [38]. Functionally, aqueous and ethanolic extracts of white mulberry fruits and leaves inhibit the growth of *V. cholerae* in vitro, producing clear inhibition zones and bacteriostatic/bactericidal concentrations that correlate positively with total phenolic and flavonoid content [29,39]. These extracts also exhibit broad-spectrum activity against *Escherichia coli*, *Salmonella Typhi*, *Shigella dysenteriae* and *Staphylococcus aureus* [29,40], which is consistent with the general antimicrobial profile of mulberry polyphenols.

MA, a plant belonging to the family Moraceae, is widely cultivated in Asia for its leaves (the main food source of silkworms) and has a long history of use in traditional medicine, where preparations from its leaves, fruits and root bark have been employed for the management of metabolic, hepatic and inflammatory disorders [41,42,43]. Recent studies have evaluated the capacity of extracts obtained from its leaves or fruits to positively modulate physiological parameters in fish, such as growth, immune response and histological integrity of key organs like the liver and intestine [32,44,45]. Specifically, in *Oreochromis niloticus*, dietary supplementation with MA leaves has been shown to significantly improve the expression of immune genes (IL-1β, IL-8, IFN-γ) and resistance against pathogens such as *Aeromonas hydrophila* [33]. In addition, the extract promotes an increase in circulating leucocytes, immunoglobulins and serum bactericidal activity [46]. Another documented effect is the stimulation of digestive enzymes such as amylase, lipase and trypsin, as well as hepatic transaminases such as ALT and AST, suggesting better nutrient utilisation and improved liver health [34,47,48].

Another relevant aspect is the capacity of MA extract to mitigate oxidative damage induced by environmental stress, such as hypoxia–reoxygenation, by suppressing the production of reactive oxygen species (ROS) and increasing the activity of antioxidant enzymes (GPx, GR, GST) [45,47]. In studies with carp and tilapia, the inclusion of aqueous or ethanolic extracts of MA has produced improvements in hepatic and splenic somatic indices, as well as in intestinal morphology, evidenced by the elongation of villi and an increase in the number of goblet cells [46]. From a histopathological perspective, the extract has been shown to preserve the tissue architecture of target organs, reducing signs of necrosis, vacuolisation and atrophy in fish exposed to stress factors or infectious agents [32,47]. This positions it as an ideal candidate for improving animal welfare and physiological resilience under intensive culture conditions.

The integration of functional additives into formulated diets constitutes an efficient and sustainable approach to improve fish performance and health without resorting to synthetic antimicrobials [18,48]. The use of MA aligns with modern aquaculture trends towards more ecological, safe and economically viable practices. However, there are no studies in *D. latifrons* that evaluate MA extract against *V. cholerae* with a pre/post-challenge approach and multiscalar read-outs, which represents a relevant gap.

Given the multifunctional potential of MA and the urgent need for prophylactic alternatives against infections such as those caused by *V. cholerae*, the present study posits the hypothesis that dietary supplementation with MA extract will improve performance, modulate biochemical and enzymatic profiles, reinforce haemato-immunology and preserve tissue integrity, thereby increasing resistance to infection in *D. latifrons*.

Therefore, the protective role of MA extract against *V. cholerae* was evaluated in vitro by characterizing *V. cholerae* growth, virulence-associated gene expression and morphological alterations in planktonic cells, and in vivo through its impact on growth, biochemical and enzymatic responses, haemato-immunology and tissue histopathology of *D. latifrons* before and after the challenge.

## 2. Materials and Methods

### 2.1. Study Site

The trial was conducted indoors at the Experimental Aquaculture Laboratory of the Universidad Técnica Estatal de Quevedo (UTEQ), located in Quevedo, Los Ríos Province, Ecuador. The site is positioned at 01°06′13″ S, 79°29′22″ W, at an elevation of 73 m above sea level.

### 2.2. Preparation of MA Extract

Leaf-shoots of MA were harvested during the active vegetative phase, 40–45 days after budburst, in accordance with recommended ‘leaf-shoot’ harvesting practices [49]. Freshly collected leaves were carefully rinsed with distilled water to remove surface impurities and dried in a forced-air oven at 40 °C for 72 h to preserve thermosensitive bioactive compounds. Fully dehydrated leaves were then milled to a fine powder using a laboratory grinder [50,51].

The powdered material was macerated in a hydroalcoholic solution of 96% ethanol and distilled water at a 7:3 (*v*/*v*) ratio. Maceration proceeded for 7 days at ambient temperature (25 ± 2 °C) in sealed amber-glass containers to minimise photodegradation [50]. The mixture was agitated daily to enhance solvent penetration and compound extraction. After maceration, the solution was filtered through standard Whatman No. 1 filter paper (Whatman, Maidstone, UK) to remove coarse plant residues. A second filtration step was performed under vacuum to increase clarity and reduce residual particulates. The filtrate was concentrated under reduced pressure in a rotary evaporator (Heidolph, Schwabach, Germany) at 40 °C to remove ethanol and obtain a semi-solid crude extract [52]. The final product was stored in sterile amber vials at 4 °C until incorporation into the experimental diets.

### 2.3. Phytochemical Profile of MA Extract

Qualitative screening of secondary metabolites in the hydroalcoholic MA extract followed standard colourimetric and precipitation reactions [53,54]. Coumarins were assessed by alkaline visualisation under UV (365 nm) after exposure to ammonia vapour (NH_3_). Anthraquinones were evaluated by the Bornträger reaction. Phenolic compounds were screened using ferric chloride (FeCl_3_), while reducing sugars were detected by Fehling’s test. Triterpenes and steroids were identified with the Liebermann–Burchard reaction. Tannins were determined by gelatine/NaCl precipitation. Flavonoids were detected using the Shinoda test. Oils and fats were investigated with the Sudan test. Alkaloids were examined with Dragendorff’s, Mayer’s, and Wagner’s reagents. Free amino acids were evaluated separately by the ninhydrin reaction.

All assays were performed in triplicate (*n* = 3) using controlled volumes of concentrated extract and reagent according to the respective protocols. A solvent blank (ethanol:water, 7:3 *v*/*v*) was run in parallel and yielded negative results in all tests. Results of the qualitative screening are presented in Table 2.

### 2.4. In Vitro Antimicrobial Susceptibility Assay

The in vitro antimicrobial susceptibility assay employed the disc diffusion method on Mueller–Hinton agar (MHA) supplemented with 1–2% NaCl, following CLSI recommendations [36]. A pure *V. cholerae* O1 strain from the UTEQ Microbiology Laboratory culture collection was used. After reactivation in Nutrient Broth (NB) for 6–8 h at 35 °C, the strain was streaked on TCBS (Thiosulfate–Citrate–Bile–Sucrose) agar to confirm purity, then cultured on nutrient agar for 18–24 h at 35 °C to obtain fresh colonies. A bacterial suspension adjusted to the 0.5 McFarland standard (1.0 × 10^8^ CFU/mL) was prepared from these colonies and uniformly spread over plate surfaces using a sterile swab.

Different concentrations (12.50, 25, 50 and 100 mg/mL) of the MA plant extract [55,56], were prepared from a stock solution, and 10 µL were applied to sterile 6-mm paper discs. The impregnated discs were then allowed to dry under sterile conditions to ensure partial solvent evaporation [51]. Commercial tetracycline discs (30 µg) served as the positive control, and discs impregnated with the same volume of ethanol were used as the negative control [57].

Discs were placed carefully on the inoculated agar surface, maintaining a minimum centre-to-centre distance of 24 mm to avoid halo overlap. Plates were incubated inverted at 35 °C for 24 h under aerobic conditions [57]. After incubation, inhibition zones were measured with a millimetre-graduated calliper, and results were reported as mean ± standard error from four independent replicates.

### 2.5. Determination of the Minimum Inhibitory Concentration (MIC)

The MIC of the MA extract against *V. cholerae* was determined using the broth microdilution method following CLSI M07-A11 guidelines [58]. Serial two-fold dilutions of the extract were prepared in a 96-well microplate using Mueller–Hinton broth as the test medium, generating final concentrations ranging from 1000 to 1 µg/mL. Each well received 50 µL of the diluted extract and 50 µL of a bacterial suspension adjusted to 5 × 10^5^ CFU/mL, in accordance with standard MIC-testing recommendations [59]. Blank wells (medium only) and growth controls (bacteria without extract) were included. Plates were incubated at 37 °C for 18 h, and bacterial growth was quantified spectrophotometrically at 600 nm (OD_600_), using blank-corrected readings [59,60].

The MIC was defined as the lowest concentration at which the OD_600_ was ≤0.05 after blank correction, indicating absence of detectable growth [60]. According to this criterion, the MIC and ½ MIC values were 250 µg/mL and 125 µg/mL, respectively, which were subsequently used as inhibitory and sub-inhibitory levels in the growth-kinetics and SEM analyses.

### 2.6. Bacterial Growth Kinetics

A single colony of *V. cholerae* grown on TCBS agar was transferred to 5 mL of LB broth and incubated at 37 °C for 18 h at 180 rpm to obtain a culture in the stationary phase, thereby avoiding the accelerated growth associated with enrichment media [61]. This primary culture was diluted 1:100 into fresh LB and incubated under the same conditions until reaching an OD_600_ of 0.12, corresponding to an initial density of 10^6^ CFU/mL. This standardized suspension was used as the inoculum for the kinetic assay.

The assay was conducted in a sterile 96-well microplate, where each well received 198 µL of standardized culture and 2 µL of the corresponding treatment (final volume 200 µL), maintaining a 1% inoculum proportion to ensure a defined lag phase. Four experimental conditions were evaluated: a growth control, a solvent control containing 1% ethanol (*v*/*v*), a sub-inhibitory MA extract treatment (½ MIC, 125 µg/mL), and an inhibitory treatment (MIC, 250 µg/mL) [62]. All treatments were tested in triplicate. Plates were incubated in a microplate reader at 37 °C programmed to record OD_600_ every 30 min for 24 h, with automatic orbital shaking prior to each measurement.

### 2.7. Culture, Standardisation and Preparation of the Planktonic Inoculum

Planktonic cultures of *V. cholerae* were obtained from a single colony on TCBS agar transferred to 5 mL of LB broth and incubated at 37 °C for 18 h at 180 rpm. The resulting culture was reinoculated (1:100) into fresh LB medium and incubated under the same conditions until reaching mid-log phase (OD_600_ = 0.12). Cell density at this optical range corresponds to approximately 1 × 10^6^ CFU/mL, matching the standardisation applied for the MIC and growth-kinetics assays. This standardized suspension was used immediately for SEM treatments [63].

For each experimental unit, 990 µL of standardized culture were mixed with 10 µL of the corresponding treatment, maintaining a final treatment proportion of 1% (*v*/*v*) consistent with the kinetic assay. Negative controls received sterile LB medium, solvent controls received ethanol at 1% (*v*/*v*), and treatments consisted of Morus alba extract at ½ MIC or MIC, as previously determined. Mixtures were gently homogenised and incubated at 37 °C for 1 h prior to SEM processing, allowing the evaluation of early morphological responses under sub-inhibitory and inhibitory conditions.

### 2.8. SEM-Based Evaluation of Bacterial Morphological Damage

Following treatment of the standardized mid-log culture (OD_600_ = 0.12), suspensions were centrifuged at 5000× *g* for 10 min, and pellets were washed twice with PBS (0.01 M, pH 7.4). Cells were fixed in 2.5% glutaraldehyde for 12 h at 4 °C, washed, and post-fixed in 1% osmium tetroxide for 1 h. Samples were dehydrated through a graded ethanol series (30–100%) and dried using critical-point drying. Dried samples were mounted on aluminium stubs with carbon tape and coated with a 7–12 nm Au/Pd layer by sputtering. Imaging was performed in secondary electron mode at 5–10 kV, working distance 8–12 mm, and magnifications of 5–30 kX.

### 2.9. Expression of Virulence-Related Genes

*V. cholerae* cultures were grown under the same conditions described for the growth-kinetics assay, using standardized mid-log suspensions (OD_600_ = 0.12 in LB), and exposed for 1 h to four treatments: extract-free control, solvent control (1% ethanol), sub-inhibitory extract (½ MIC, 125 µg/mL) and inhibitory extract (MIC, 250 µg/mL). RNA was extracted using a bacteria-specific kit (QIAGEN, Hilden, Germany), eluted in 50 µL nuclease-free buffer, treated with DNase I, and assessed for purity (A260/280 = 1.9–2.1). RNA integrity was verified by agarose gel electrophoresis showing well-defined 23S/16S rRNA bands. RT controls were included to confirm absence of genomic DNA contamination in accordance with MIQE guidelines [64].

cDNA synthesis was performed from 1 µg total RNA using a bacterial RNA-compatible reverse transcription kit (QIAGEN, Hilden, Germany) with random hexamers (and oligo-dT when recommended). cDNA was diluted 1:10 before qPCR. Reactions (20 µL) contained 10 µL 2× SYBR Green Master Mix (QIAGEN, Hilden, Germany), 0.4 µL of each primer (10 µM; final 0.2 µM), 2 µL cDNA, and nuclease-free water. The selected targets, *toxR* and *ompU*, represent key regulators and effectors of virulence and stress response, while *recA* served as the reference gene due to its transcriptional stability [65]. Primer sequences and amplicon lengths are listed in Table 1. The cycling protocol consisted of 95 °C for 3 min, followed by 40 cycles of 95 °C for 10 s and 60 °C for 30 s, with a melt-curve analysis to confirm specificity. Relative expression was quantified using the 2^−ΔΔCt^ method with the extract-free control as calibrator and *recA* as reference gene [64,65].

### 2.10. Incorporation of the Extract into Experimental Diets for Fish

Five dietary treatments were formulated by incorporating MA extract at 0 (control), 5, 10, 15 and 20 g/kg of commercial feed. The basal diet for all treatments was a commercial pellet (Aquachampion^®^, Guayaquil, Ecuador) containing 35% crude protein and 5% lipids. Inclusion levels were selected on the basis of previous experimental findings and dose–response studies reported by Sheikhlar et al. [45] and Tang et al. [46].

To ensure uniform distribution of the extract, commercial pellets were first ground to a fine powder with particle sizes < 250 µm. Each diet was prepared using 1% food-grade gelatine as a binder, 35% warm water (approximately 40 °C), and the corresponding concentration of MA extract. The mixture was thoroughly homogenised and extruded through a meat grinder fitted with a 2-mm die to produce uniform pellets. Freshly extruded diets were oven-dried at 45 °C for 8 h to reduce moisture content and ensure pellet stability, following published protocols [46]. Once dried, pellets were placed in sterile plastic bags and stored at 4 °C until feeding.

### 2.11. In Vivo Experimental Design and Rearing Conditions

Juvenile *D. latifrons* (mean body mass 10.06 ± 0.50 g) were used. Prior to the feeding trial, fish underwent a 7-day acclimatisation period under controlled laboratory conditions. Following acclimatisation, fish were randomly allocated to 15 plastic aquaria (three replicates per dietary treatment), each with a working volume of 50 L and stocked at a density of 15 fish per aquarium. The feeding trial lasted for eight consecutive weeks, after which all groups were subjected to a 7-day bacterial challenge with *V. cholerae.*

Throughout the trial, fish were offered their respective experimental diets to apparent satiation twice daily, at 09:00 and 17:00 h. Feeding was adjusted weekly based on biomass to minimise feed waste. Uneaten feed, identifiable by its shape and texture, was collected the following morning. Leftover feed was retrieved using Whatman No. 1 filter paper and a vacuum pump (KNF Neuberger, Freiburg, Germany), then oven-dried at 50 °C for 18 h to quantify feed intake [69].

Each morning, all tanks were siphoned to remove faeces and any uneaten feed prior to the first feeding, and the working volume was restored with clean, oxygenated water. Natural photoperiod conditions were maintained throughout (12 h light:12 h dark). Water quality was monitored daily. Dissolved oxygen was measured with a digital oximeter; water temperature was measured with a standard mercury thermometer (0–50 °C range); and nitrate (NO_3_^−^), ammonium (NH_4_^+^), pH, and carbonate concentrations were determined using a colourimetric test kit (API, Chalfont, PA, USA). During the trial, water quality remained within optimal ranges for the species: dissolved oxygen 5.58 ± 0.10 mg/L, temperature 28.5 ± 0.50 °C, nitrate 0.46 ± 0.30 mg/L, ammonium 0.04 ± 0.02 mg/L, pH 7.3 ± 0.07, and carbonates 4.2 ± 1.50 mg/L.

### 2.12. Sample Collection from Fish

At the end of the eight-week feeding trial, all fish were fasted for 24 h and then anaesthetised in an aqueous eugenol solution at 40 mg/L. Each fish was weighed on a precision digital balance (±0.01 g) and total length was measured with a digital calliper (±0.001 mm). Subsequently, five juveniles were randomly selected from each tank (*n* = 15 per treatment group) and blood was collected by caudal venepuncture at the level of the haemal arch using sterile disposable 1-mL syringes. Blood for haematology was kept at 4 °C until processing (<6 h). Blood for biochemical analyses was centrifuged at 3000 rpm for 10 min at 4 °C, and the resulting plasma was stored at −20 °C until analysis.

Following blood collection, the same fish were euthanised with an overdose of eugenol (80 mg/L). Death was confirmed by the absence of opercular movements, loss of the righting reflex, and lack of response to tactile and ocular stimuli. Fish were carefully eviscerated and the mid-intestine, liver, and spleen were dissected and immediately fixed in 10% neutral-buffered formalin for 24 h for subsequent histological analysis. Eviscerated fish were placed in airtight polyethylene bags (SC Johnson, Racine, WI, USA) and stored at −20 °C for later determination of whole-body proximate composition (eviscerated). The same sampling procedures were applied to fish collected at the end of the post-challenge period with *V. cholerae* (day 7).

### 2.13. Growth Performance and Feed Utilisation

Mathematical equations were used to determine specific growth rate, weight-gain rate, feed conversion ratio, condition factor, survival rate, and the relative level of protection, as detailed below [69]:

Weight-gain rate (WGR, %) = [Wx − Wi] × 100.

Specific growth rate (SGR, % day^−1^) = 100 × [(lnWx − lnWi)/t].

Condition factor (K) = 100 × [Wx/Lx^3^].

Feed conversion ratio (FCR) = total feed consumed (g, dry weight)/total weight gain (g, wet weight).

Feed efficiency (FE) = total weight gain (g, wet weight)/total feed consumed (g, dry weight).

Protein efficiency (PE) = Protein intake/total feed consumed (g, dry weight).

where t is the duration of the experiment (days), Lx is final body length (cm), Wx is final body weight (g), and Wi is initial body weight (g).

### 2.14. Whole-Body Proximate Composition

The proximate composition of eviscerated whole fish was determined on a wet-weight basis following standard procedures of the Association of Official Analytical Chemists [70]. Moisture content was assessed by drying samples in a convection oven at 105 °C to constant weight. Ash content was measured by incineration in a muffle furnace at 550 °C for 8 h. Crude protein was estimated using the Kjeldahl method with a nitrogen-to-protein conversion factor of 6.25. Lipid content was determined by ether extraction using a Soxtec system (FOSS Tecator, Hillerød, Denmark). All analyses were performed in triplicate.

### 2.15. Plasma Biochemistry

Plasma biochemical parameters were determined using LiquiColor^®^ commercial kits (Diagnostics GmbH, Wiesbaden, Germany), following the manufacturer’s instructions and in triplicate. All reactions were incubated at 37 °C and absorbance was measured on a semi-automatic spectrophotometer (BioSystems S.A., Barcelona, Spain) with appropriate wavelength calibration. Total protein (g/dL) was quantified by the biuret method (546 nm, after 10 min incubation); albumin (g/dL) by the BCG dye-binding method (630 nm, 5 min); glucose (mg/dL) by GOD-PAP (500 nm, 10 min); triglycerides (mg/dL) by the GPO-PAP method (546 nm, 10 min); and total cholesterol (mg/dL) by CHOD-PAP (500 nm, 10 min). Urea (mg/dL) was evaluated by the urease–GLDH method (340 nm, 5 min), while creatinine (mg/dL) was measured using the kinetic Jaffé method with dual readings at 492 nm after 30 and 120 s, respectively [71,72].

### 2.16. Digestive, Metabolic and Antioxidant Enzymes

Activities of aspartate aminotransferase (AST) and alanine aminotransferase (ALT) were determined using LiquiColor^®^ commercial kits (Diagnostics GmbH, Wiesbaden, Germany). AST and ALT activities were measured according to the kinetic method of Bergmeyer et al. [73], based on the oxidation of NADH to NAD^+^, with absorbance recorded at 340 nm. Samples were incubated at 37 °C for 15 min (AST) and 5 min (ALT), respectively, and absorbance was monitored for 3 min. Enzyme activities were expressed as units per litre (U/L).

Activities of amylase and lipase were measured in plasma by colourimetric kinetic assays using commercial kits (Spinreact S.A.U., Girona, Spain), following the manufacturer’s instructions. α-Amylase was assayed by monitoring the hydrolysis rate of a chromogenic substrate at 37 °C, recording the increase in absorbance at 405 nm for 3 min. Lipase activity was determined using a synthetic ester substrate that releases a measurable chromophore; reactions were run at 37 °C and the increase in absorbance at 580 nm was monitored for 3 min. Enzyme activities were expressed in U/L.

Antioxidant enzyme activities were determined by colourimetric kinetic assays using commercial reagents (Randox Laboratories Ltd., Crumlin, UK) and standard methodologies. Superoxide dismutase (SOD) activity was assayed by inhibition of the reduction of iodonitrotetrazolium chloride (INT) by superoxide radicals generated via the xanthine/xanthine-oxidase system; absorbance was read at 505 nm [74,75]. Glutathione peroxidase (GPx) activity was quantified by monitoring the decrease in NADPH at 340 nm in the presence of reduced glutathione, cumene hydroperoxide, and glutathione reductase [76]. Catalase (CAT) activity was determined by the Aebi spectrophotometric method [77], based on the decomposition of hydrogen peroxide; absorbance at 240 nm was monitored at 25 °C for 3 min. Enzyme activities were expressed as U/mL. All measurements were performed in triplicate to ensure analytical precision and reproducibility.

### 2.17. Haematoimmunology

Haemoglobin concentration (g/dL) was determined using Drabkin’s reagent according to the cyanomethaemoglobin method, with absorbance measured at 546 nm after a 10-min reaction time. Total erythrocyte (×10^12^/L) and leucocyte (×10^9^/L) counts were performed using a Neubauer haemocytometer (Brand GmbH, Wertheim, Germany; 0.0025 mm^2^). Hayem’s solution was used as the diluent for erythrocyte counts, while an acetic acid-based reagent was used for leucocyte quantification. In both cases, samples were diluted at 1:200. Haematocrit (%) was measured using a microcapillary centrifuge at 10,000 rpm for 5 min. Erythrocyte indices: mean corpuscular volume (MCV, fL), mean corpuscular haemoglobin (MCH, pg), and mean corpuscular haemoglobin concentration (MCHC, g/dL) were calculated using standard formulae based on red blood cell count, haemoglobin concentration, and haematocrit. Procedures followed Blaxhall and Daisley [78], Hrubec and Smith [79]. All analyses were conducted in triplicate.

### 2.18. Histomorphology

Samples of intestine, liver, and spleen were fixed in 10% neutral-buffered formalin. Tissues were then dehydrated through a graded ethanol series (70% to 100%), embedded in paraffin, and sectioned at 5 µm using a rotary microtome (Leica Microsystems, Wetzlar, Germany). All samples were stained with haematoxylin and eosin (H&E) for general histological assessment. Periodic acid–Schiff (PAS) staining was additionally applied to liver, while a combined Alcian blue–PAS protocol was used specifically for intestinal sections.

Histological sections were examined with a light microscope equipped with a colour digital camera. Images were analysed using Image Scion 4.0.2 software, with which morphometric evaluations were performed.

### 2.19. Bacterial Challenge-Induced Stress

After eight weeks of dietary treatment, 24 individuals per group (*n* = 24) were challenged by intraperitoneal injection with the *V. cholerae* strain previously described in the antibiogram section. Bacteria were cultured on TCBS agar for 24 h at 35 °C and subsequently amplified for 8 h in alkaline peptone water (APW). The inoculum was centrifuged at 3000× *g* for 10 min, and the bacterial pellet resuspended in sterile PBS (pH 7.4). Concentration was adjusted spectrophotometrically at OD_600_ to 1.5 × 10^6^ CFU/mL and validated by plate counts and each fish received an intraperitoneal dose proportional to body mass at 0.15 mL/10 g body weight [80]. Prior to injection, fish were anaesthetised with eugenol.

Following inoculation, fish in each replicate were maintained under controlled, optimal water-quality parameters and photoperiod. Fish were monitored daily for clinical signs and mortality over seven days. Dead fish were removed immediately for necropsy, and cause of death was confirmed as *V. cholerae* infection by isolating colonies on TCBS agar and observing characteristic morphology. Survival rate and relative level of protection (RLP) were calculated using the formula of Ruangpan et al. [81].

Survival Rate (%) = 100 × [final number of fish/initial number of fish]

Relative level of protection (RLP, %) = [1 − (% mortality of treated fish/% mortality of control fish)] × 100.

### 2.20. Statistical Analysis

Data normality was first assessed using the Kolmogorov–Smirnov test and homoscedasticity with Levene’s test to confirm compliance with parametric assumptions. Variables that did not meet assumptions were transformed using the arcsine square-root transformation $\left[y^{\prime }=arcsin(\sqrt{p})\right]$. Thereafter, a one-way ANOVA was applied, followed by Fisher’s least significant difference (LSD) test to identify significant differences among treatments (*p* < 0.05).

These analyses were performed in Minitab^®^ 19 (Minitab LLC, State College, PA, USA). All data were standardised by Z-score transformation (mean = 0; standard deviation = 1) to homogenise scale and variance among variables. From the standardised matrix, a heat map (Ward’s linkage, Euclidean distance) was generated, together with a principal component analysis (PCA) to explore multivariate structure among treatments. These analyses were conducted in RStudio (v. 2023.06.1+524, RStudio, Boston, MA, USA) using the Pheatmap and FactoMineR packages.

## 3. Results

### 3.1. Phytochemical Screening and Antibiogram Test

Table 2 summarises the qualitative reactions for the hydroalcoholic extract of MA. Intense positivity (+++) was observed for triterpenes/steroids (Liebermann–Burchard) and for flavonoids (Shinoda), together with a marked positivity for alkaloids confirmed by Wagner (+++) and additional positivity with Dragendorff (+), whereas Mayer was negative. Moderate positivity (++) was also recorded for anthraquinones (Bornträger reaction). Total phenolics (FeCl_3_) showed low positivity (+) and, where the specific test was applied, tannins yielded a positive signal (+) with gelatine/NaCl. Among primary metabolites, reducing sugars (+) (Fehling) and free amino acids (+) (ninhydrin) were detected. In contrast, coumarins assessed by UV fluorescence after exposure to NH_3_ vapour and oils/fats (Sudan) were negative (–). Overall, the profile indicates a predominance of triterpenes/steroids, alkaloids and flavonoids, with concomitant detection of anthraquinones and phenolics, and absence of coumarins and dyeable lipid fractions.

Table 3 presents the inhibition zone diameters of *V. cholerae* in response to extract of MA. A clear dose-dependent increase was observed, from 7.12 mm at 12.5 mg/mL to 13.93 mm at 100 mg/mL. The positive control (tetracycline, 30 µg) yielded a zone of 18.30 mm, whereas the negative control (ethanol) showed no inhibition. Although the inhibition zones produced by the crude MA extract were smaller than those obtained with tetracycline, the observed response demonstrates consistent and concentration-dependent antibacterial activity against *V. cholerae*, which is compatible with the expected potency profile of a crude plant extract when compared with a reference antibiotic.

### 3.2. Growth Kinetics, Virulence Gene Expression and Morphological Alterations

**Figure 1:**
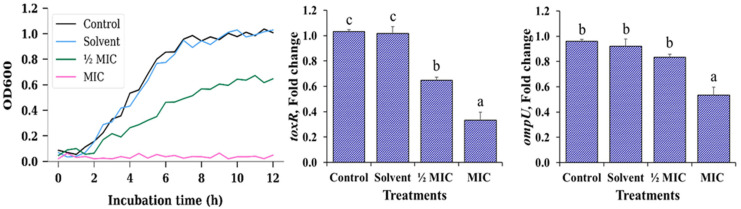
Growth kinetics and relative expression of the virulence-related genes *toxR* and *ompU* in *V. cholerae* under different treatments with *Morus alba* (MA) extract. For clarity, only data from the first 12 h of the growth kinetics are shown, as extended monitoring up to 24 h did not reveal further changes in growth patterns. Treatments: negative control (no solvent, no extract), solvent control (1% ethanol), ½ MIC (125 µg/mL) MA extract, MIC (250 µg/mL) MA extract. Data are expressed as mean ± standard error (*n* = 3 per treatment). Different letters (a–c) indicate significant differences among treatments (*p* < 0.05).

In the growth kinetics panel of Figure 1, *V. cholerae* exhibited a very similar pattern between the negative control and the solvent control (1% ethanol), with a short lag phase followed by a pronounced exponential phase during the first hours of incubation. In both cases, cultures reached maximum OD_600_ values close to 1.0 before entering the stationary phase, indicating that ethanol, at the concentration used, does not exert a detectable effect on bacterial proliferation or on the overall growth dynamics. This validates the use of 1% ethanol as an appropriate vehicle for the MA extract in subsequent assays.

In contrast, exposure to the *Morus alba* extract at ½ MIC clearly modified the kinetic profile. The curve displayed a reduced slope during the exponential phase and a lower plateau in the stationary phase, reflecting a decrease in both growth rate and final biomass. This behaviour is consistent with a predominantly bacteriostatic effect, where cells remain viable but their capacity to multiply is partially restricted in the presence of sub-inhibitory concentrations of the extract. At the MIC, OD_600_ values remained close to the basal level throughout the 12 h observation period, with no evidence of a defined exponential phase. This flat profile indicates complete suppression of detectable growth under these conditions, suggesting that the extract, at this concentration, is capable of fully preventing the expansion of the planktonic population, even though the available data do not allow a distinction between a strictly bacteriostatic or bactericidal action.

The analysis of virulence gene expression (Figure 1) revealed a concentration-dependent transcriptional response to the MA extract. Expression of *toxR* remained close to unity in both the negative control and the solvent control, confirming that the vehicle alone does not interfere with the basal activity of this master regulator. However, at ½ MIC, *toxR* showed a moderate yet consistent decrease, and at MIC a marked downregulation was observed, with statistically significant differences relative to the control (*p* < 0.05). This pattern suggests that *toxR* is sensitive to the stress induced by the extract even at sub-inhibitory levels, indicating an early anti-virulence effect in addition to its impact on growth.

For *ompU*, the control, solvent and ½ MIC treatments maintained relative expression values close to 1, with no significant differences among them, indicating that moderate exposure to the extract does not appreciably alter the transcription of this outer membrane porin. Only the MIC induced a significant reduction in *ompU* expression (*p* < 0.05), an effect that became evident solely under fully inhibitory conditions. Taken together, these findings indicate that the MA extract not only limits the proliferation of *V. cholerae* in a concentration-dependent manner, but also modulates its virulence potential. *toxR* appears to respond earlier, even at sub-inhibitory concentrations, whereas significant repression of *ompU* is only detected when bacterial growth is completely inhibited, suggesting a hierarchical or sequential sensitivity of these virulence-associated targets to the extract.

**Figure 2:**
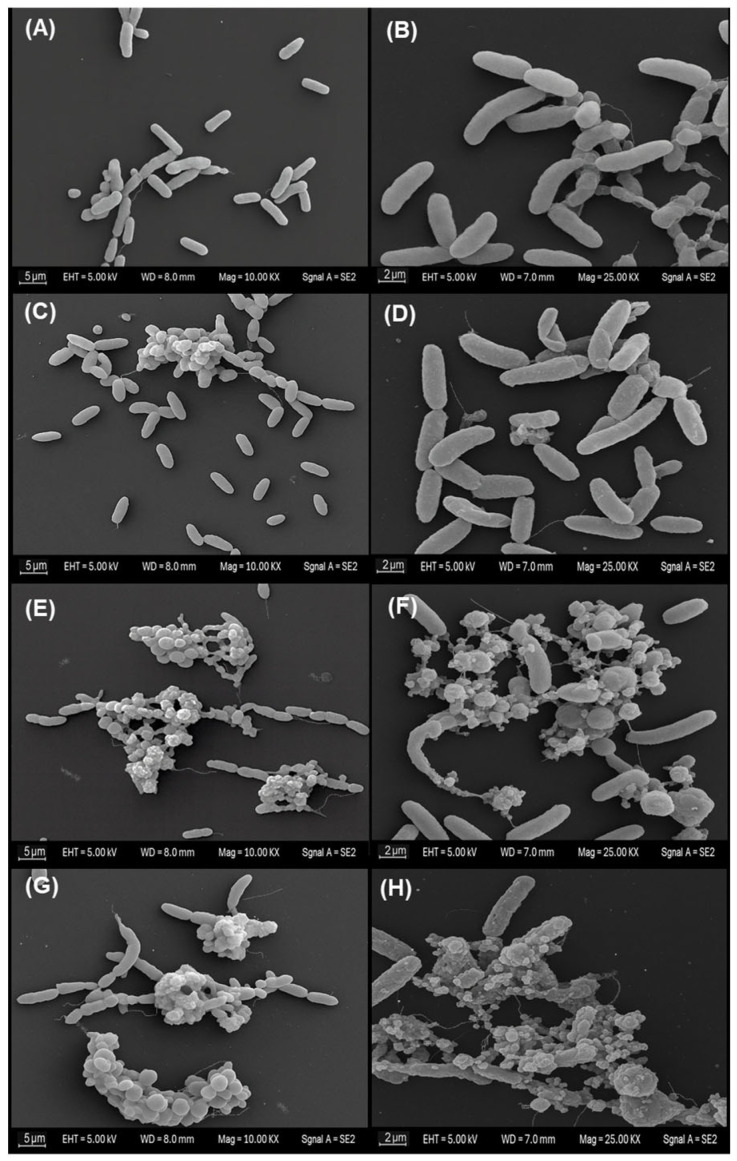
Scanning electron micrographs showing concentration-dependent morphological changes in planktonic *V. cholerae* exposed to MA extract. (**A**,**B**) Negative control. (**C**,**D**) Solvent control (1% ethanol). (**E**,**F**) ½ MIC (125 µg/mL). (**G**,**H**) MIC (250 µg/mL). Images represent three independent biological replicates (*n* = 3).

Scanning electron microscopy of planktonic *Vibrio cholerae* exposed to the MA extract revealed a clear concentration-dependent gradient of morphological alterations across treatments (Figure 2). In the negative control (panels A and B), bacteria retained the characteristic bacillary morphology, with well-defined contours, smooth and continuous surfaces, and no signs of envelope collapse. Cells appeared predominantly dispersed, with minimal aggregation and an absence of amorphous extracellular material; this condition was therefore considered the basal morphological reference. Similarly, in the solvent control (panels C and D), cells preserved an appearance comparable to the negative control, displaying intact rod-shaped forms, homogeneous surface topography and apparently continuous membranes. Only occasional microaggregates and a slight increase in the proximity between cells were observed, without noticeable deformation or accumulation of debris, indicating that ethanol at 1% (*v*/*v*) did not induce appreciable morphological changes under the assay conditions.

In contrast, exposure to the MA extract at the sub-inhibitory concentration (½ MIC; panels E and F) produced more evident modifications. Compact cellular aggregates were observed, frequently embedded within granular or amorphous material. At higher magnification, several cells exhibited irregular or distorted bacillary outlines, roughened surfaces, and areas suggestive of thinning, wrinkling or localised discontinuities in the cell envelope. These features are consistent with partial or early-stage structural damage, although without evidence of complete loss of cellular integrity across the population.

The most pronounced alterations were observed at the inhibitory concentration (MIC; panels G and H). Micrographs revealed extensive clusters of cellular remnants and abundant amorphous extracellular material. Within these aggregates, numerous bacilli showed markedly collapsed morphology, severe surface irregularities and clear envelope discontinuities. At higher resolution, several cells exhibited pronounced deformation compatible with advanced envelope disruption and possible lytic processes. Taken together, these observations suggest that the MA extract induces progressive structural impairment, culminating in extensive morphological damage at the inhibitory concentration.

### 3.3. Growth Performance and Protection Level

Table 4 shows significant differences among treatments (*p* < 0.05) in the growth performance of *D. latifrons*. Final weight, weight gain, and SGR increased with dose, reaching maxima at 20 g/kg (27.57 g; 1.80%/day). Concomitantly, FCR decreased to 1.24 at 20 g/kg. Final length was greater in the supplemented groups (maximum 137.71 mm at 20 g/kg vs. 115.51 mm in the control), whereas the condition factor was lower at the higher doses (minimum 1.06 at 20 g/kg).

**Figure 3:**
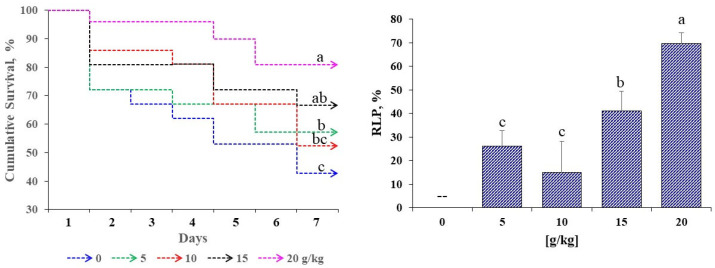
Survival and relative level of protection (RLP) in *D. latifrons* fed diets supplemented with MA extract challenged for seven days with *V. cholerae.* Results are reported as measures ± standard error of 3 groups per treatment (*n* = 3). Different letters (abc) indicate significant differences (*p* < 0.05).

Figure 3 presents the cumulative survival and RLP of *D. latifrons* following challenge with *V. cholerae*. Significant differences were detected among treatments (*p* < 0.05). Final survival increased with dose, peaking at 20 g/kg, whereas the control recorded the lowest values. RLP followed the same pattern, with a maximum at 20 g/kg, while 5 g/kg and 10 g/kg did not differ significantly from each other. The higher doses yielded the best performance in both growth and survival.

### 3.4. Proximate Composition of Eviscerated Whole-Body

Table 5 presents the proximate chemical composition after eight weeks of feeding with MA. Significant differences were observed among treatments (*p* < 0.05), except for ash (*p* > 0.05). Moisture was higher in all supplemented groups, peaking at 20 g/kg (76.36%) versus the control (73.50%). Crude protein increased with dose, reaching 21.89% at 20 g/kg compared with 16.24% in the control. Lipid content was also higher at 15–20 g/kg (5.77–5.83%) than in the control (4.91%). For ash, no significant differences were detected, although a slight upward trend was noted, with the highest value at 20 g/kg.

### 3.5. Blood Biochemical Profile

Table 6 presents the plasma analytes before and after the challenge with *V. cholerae*. Before the challenge, significant differences among treatments were detected for triglycerides, cholesterol, glucose, bilirubin and urea (*p* < 0.05), whereas creatinine did not differ (*p* > 0.05). In general, triglycerides were lower at 15–20 g/kg than in the control; cholesterol was higher at 10–15 g/kg; and glucose decreased at 10 and 20 g/kg. Urea increased at 15 g/kg. Bilirubin showed minimal variation among treatments. Plasma protein was lower in the control and higher in all supplemented groups.

After the challenge, all variables differed among treatments (including creatinine), showing dose-dependent trends (*p* < 0.05). Triglycerides and cholesterol reached maxima at 20 g/kg (followed by intermediate doses). Glucose increased in all groups relative to baseline, with the highest level at 15 g/kg. Likewise, bilirubin, urea and creatinine rose progressively with dietary inclusion. Plasma proteins showed the greatest increase at 15–20 g/kg, whereas the control exhibited the smallest change.

### 3.6. Digestive, Metabolic, and Antioxidant Enzymes

Table 7 presents the activities of digestive (amylase, lipase), metabolic (AST, ALT), and antioxidant (SOD, CAT, GPx) enzymes before and after the challenge with *V. cholerae*. Before the challenge, significant differences were detected among treatments (*p* < 0.05). AST and ALT increased at intermediate doses and were minimal at 20 g/kg. Amylase reached its maximum at 20 g/kg (with increases also at 10 g/kg), whereas lipase was higher at 10 g/kg than in the control and the extreme doses. In the antioxidant system, SOD rose gradually up to 15 g/kg, and CAT and GPx peaked at 20 g/kg; overall, 10–20 g/kg outperformed the control.

After the challenge, all enzymes differed among treatments (*p* < 0.05). AST and ALT were highest in the control and lower at 15–20 g/kg. Amylase and lipase recorded maximum values at 15–20 g/kg, exceeding the control. SOD, CAT and GPx increased following exposure, with maxima concentrated at 10–20 g/kg. Intermediate–high doses (15–20 g/kg) thus produced greater digestive and antioxidant activity and lower transaminases post-challenge.

### 3.7. Haematology

Before the challenge, haematological parameters (Table 8) varied significantly (*p* < 0.05) with MA supplementation. Haematocrit, haemoglobin and RBC reached maximum values at 20 g/kg, exceeding the control. Erythrocyte indices showed a dose-dependent effect: MCV and MCH increased at 10 g/kg, whereas MCHC showed no differences. WBC also increased significantly, with the best response at 20 g/kg, indicating haematopoietic stimulation.

After the challenge, haematocrit and haemoglobin decreased across all treatments (*p* < 0.05), although diets with 10 and 20 g/kg attenuated the reduction. RBC remained without significant differences. Erythrocyte indices varied: MCH increased at 5 and 15 g/kg, whereas MCHC was higher at 10–20 g/kg. WBC increased significantly at 15 and 20 g/kg, indicating a better cellular immune response to infection.

### 3.8. Intestinal Histopathology

**Figure 4:**
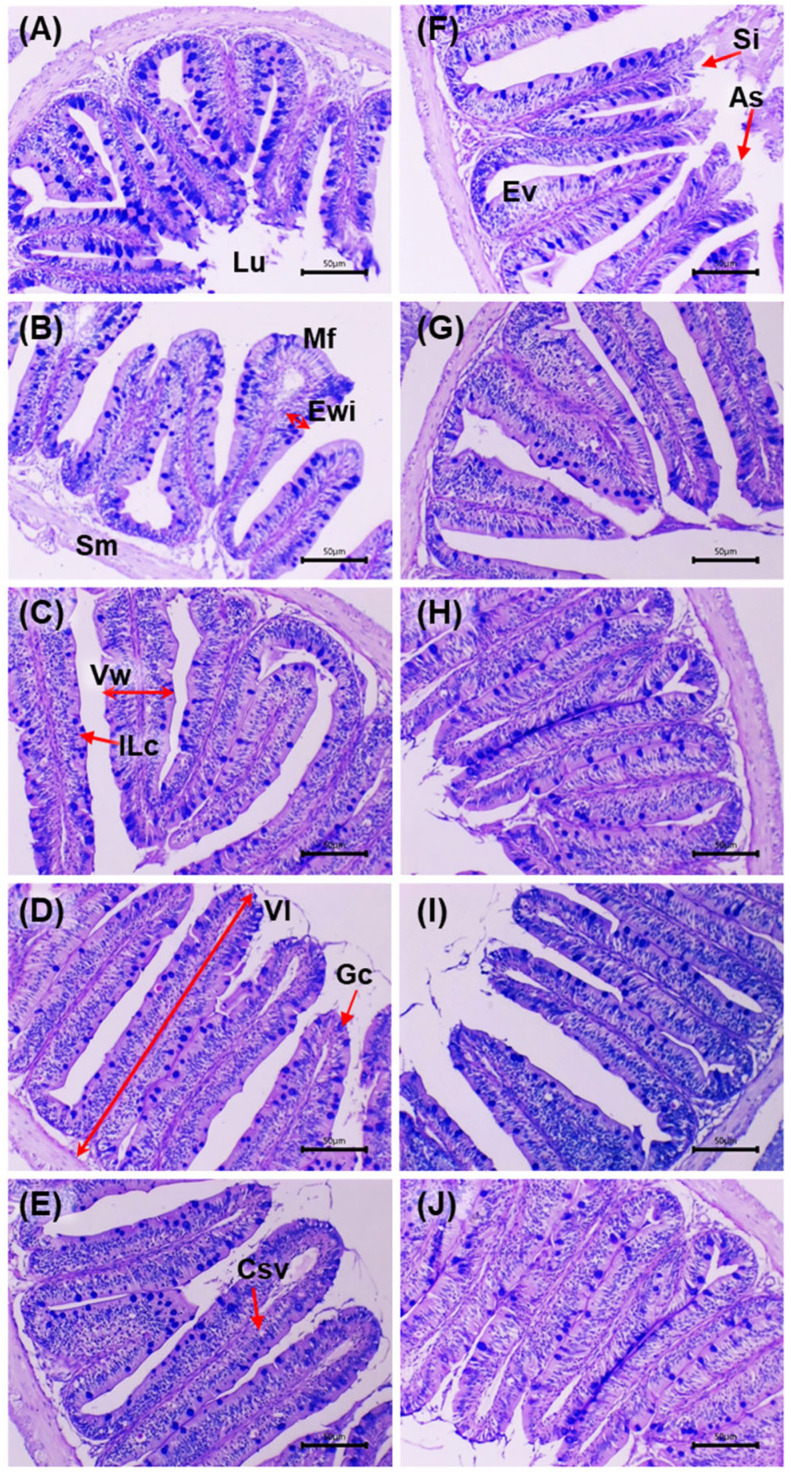
Histological micrographs of the intestine in *D. latifrons* fed diets supplemented with 0, 5, 10, 15 and 20 g/kg MA extract (**A**–**E**) for eight weeks without *V. cholerae* challenge, and of individuals challenged with the same pathogen under the same dietary treatments (**F**–**J**) for seven days, respectively. Stain: Alcian blue–periodic acid–Schiff (AB–PAS). Scale bar: 50 µm. Magnification: 10×. Abbreviations—Lu: Lumen, Sm: Subepithelial mucosa, Mf: Mucosal fold, Vw: Villus Width, Vl: Villus Length; Ewi, Enterocyte Width; Gc: Goblet Cell, Ilc: Intraepithelial Lymphocytes, Ev: Enteritic Vacuolization, CSv: Clear Supranuclear Vacuoles, Si: Subepithelial Lifting, As: Apical Sloughing.

Figure 4 shows intestinal sections stained with AB–PAS before and after the challenge with *V. cholerae*. Before the challenge, the control (0 g/kg) displayed an intact mucosa: simple columnar epithelium with well-defined basal nuclei, villi with preserved architecture and regularly distributed goblet cells; the lamina propria and submucosa without inflammatory infiltrate, and compact muscular layers. In the supplemented groups (5–20 g/kg), a trophic mucosal response was observed, with greater villus height/thickness, enterocytes with dense cytoplasm and orderly nuclei, and an increased number of goblet cells with marked AB–PAS positivity.

After the challenge, the control exhibited acute lesions: shortening and fusion of villi, epithelial necrosis, cell desquamation, disorganisation of goblet cells, inflammatory infiltrate in the lamina propria and submucosal congestion, with focal loss of epithelial continuity. In the supplemented groups, a dose-dependent preservation gradient was observed: at 5 g/kg, focal areas of epithelial degeneration and mild submucosal oedema; at 10 g/kg, partial epithelial regeneration and greater villus height with moderate lymphoid activity; at 15 g/kg, near-normal architecture of crypts, vessels and villi, minimal infiltration and intensely AB–PAS-positive mucins; at 20 g/kg, the highest degree of integrity: intact villi, active goblet cells, uncongested submucosa and preserved mucosal organisation. Histological integrity and mucin intensity showed better preservation in the supplemented treatments, with attenuation of post-challenge lesions relative to the control.

Table 9 presents the intestinal morphometric variables before and after the challenge with *V. cholerae*. Before the challenge, significant differences were observed among treatments (*p* < 0.05). The 10–20 g/kg groups showed a higher number of villi and greater villus height and width than the control. Enterocyte width increased in a dose-dependent manner, peaking at 20 g/kg. Goblet cell counts were higher at intermediate levels (peak at 15 g/kg), whereas intraepithelial lymphoid cells were more numerous at 5 g/kg, without a monotonic trend at higher doses.

After the challenge, significant differences among treatments persisted (*p* < 0.05). The supplemented diets maintained a greater number of villi than the control (maximum at 20 g/kg) and preserved villus height and width, particularly at 15–20 g/kg. Enterocyte width continued to increase with dose (maximum at 20 g/kg). The number of goblet cells was significantly higher in all extract-supplemented groups (peak at 20 g/kg), whereas intraepithelial lymphoid cells decreased with dose, with the lowest value at 20 g/kg compared with the control.

### 3.9. Liver Histopathology

**Figure 5:**
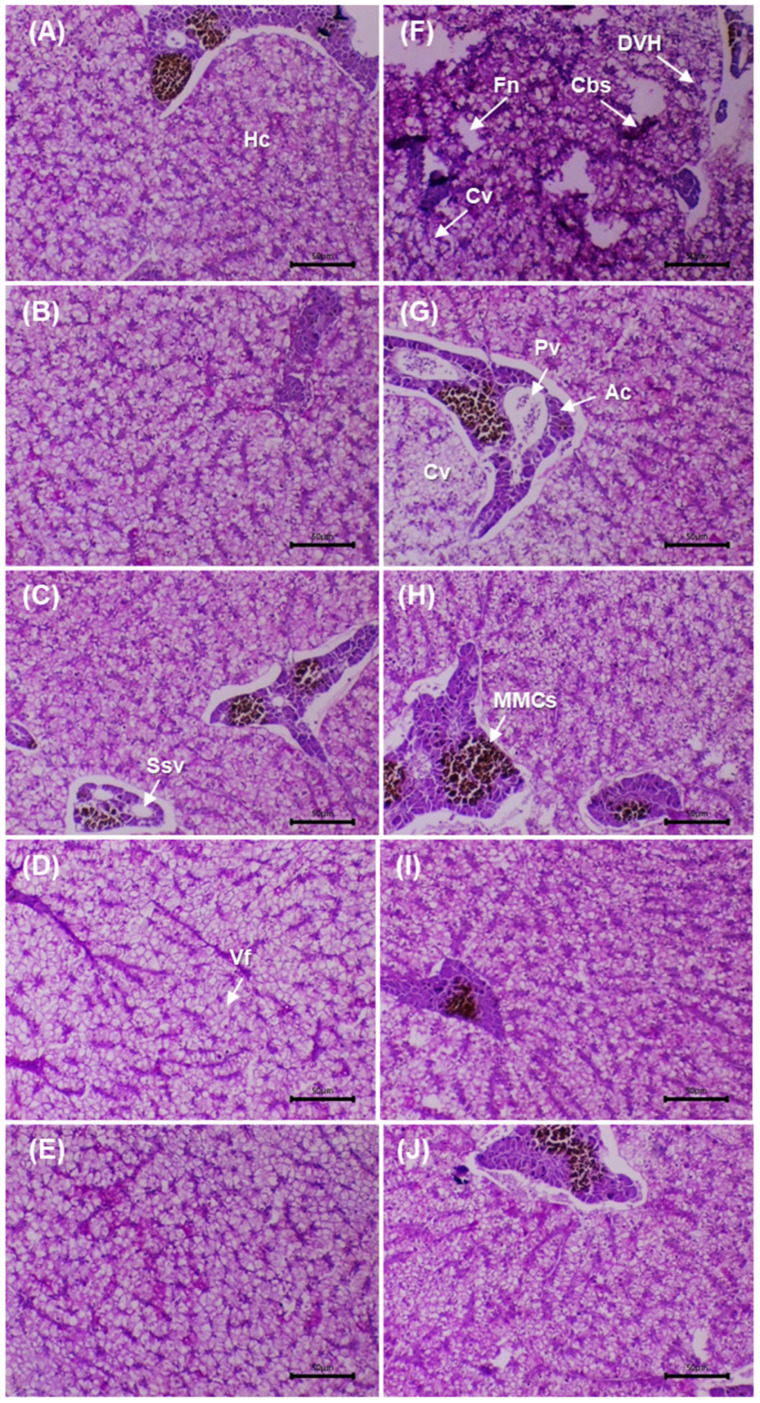
Histological micrographs of the liver in *D. latifrons* fed diets supplemented with 0, 5, 10, 15 and 20 g/kg MA extract (**A**–**E**) for eight weeks without *V. cholerae* challenge, and of individuals challenged with the same pathogen under the same dietary treatments (**F**–**J**) for seven days, respectively. Stain: periodic acid–Schiff (PAS). Scale bar: 50 µm. Magnification: 10×. Abbreviations—Ssv: Sinusoidal blood vessels, Hc: Hepatocytes, MMCs: Melanomacrophage centers, Ac: Pancreatic acinar cells organized around the vein, Cbs: Blood congestion in sinusoids, Pv: Portal vein with the presence of erythrocytes, Cv: Cytoplasmic vacuolization (hydropic degeneration), Fn: Focal necrosis, DVH: Hepatocyte vacuolar degeneration, Vf: Vacuole formation.

Figure 5 shows liver sections stained with PAS before and after the challenge with *V. cholerae*. Before the challenge, the control (0 g/kg) displayed smaller hepatocytes, pale cytoplasm and irregular PAS positivity, with mild vacuolation and slight sinusoidal congestion. In the supplemented groups (5–20 g/kg), a progressive increase in PAS positivity and cytoplasmic homogeneity was observed, with well-defined nuclei and preserved trabecular architecture. At 10–20 g/kg, greater cytoplasmic volume was evident without degenerative features, and sinusoids were permeable and well organised.

After the challenge, the infected control showed reduced PAS positivity, focal necrosis, sinusoidal congestion and periportal infiltration, consistent with acute injury. At 5 g/kg, partial recovery was evident (mild vacuolation and lower PAS). At 10 g/kg, structural restoration was observed, with well-defined hepatocytes and positive PAS. At 15–20 g/kg, normal lobular architecture was maintained, with voluminous hepatocytes, patent sinusoids and minimal inflammation. Glycogen distribution (PAS) and architectural integrity were preserved in the supplemented groups, with better conservation at 10–20 g/kg compared with the post-challenge control.

Table 10 presents the hepatic morphometric variables before and after the challenge with *V. cholerae*. Before the challenge, significant differences among treatments were observed for HA, HCA, HNA, RCN and HP (*p* < 0.05). In general, maximum values were recorded at 20 g/kg, with dose-dependent increases particularly clear in HCA, RCN and HP relative to the control. After the challenge, significant differences persisted (*p* < 0.05). The control showed marked reductions in HA and HCA, whereas the supplemented diets maintained higher values across all metrics, with peaks at 15–20 g/kg. The higher doses preserved greater cell and cytoplasmic size and an elevated RCN following exposure, compared with the control.

### 3.10. Splenic Histopathology

**Figure 6:**
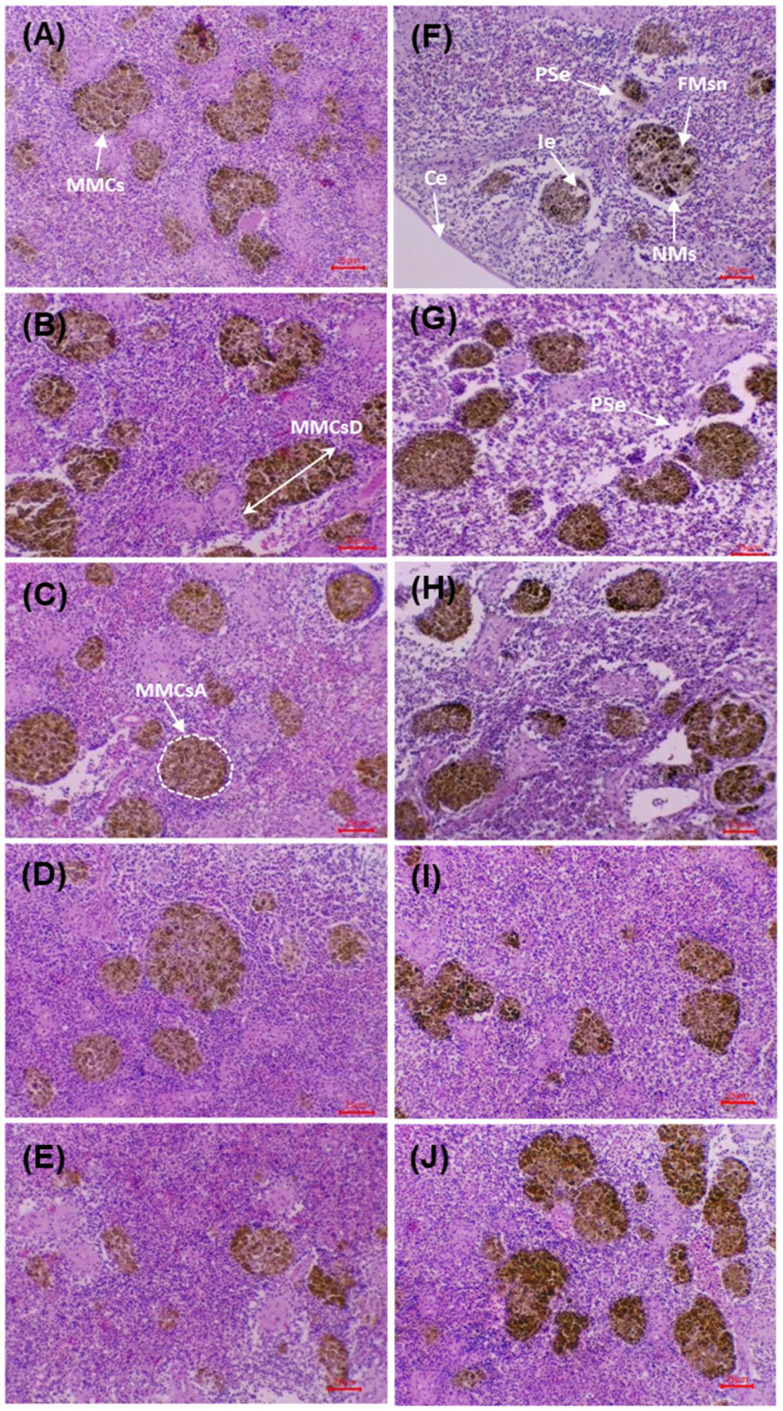
Histological micrographs of the spleen in *D. latifrons* fed diets supplemented with 0, 5, 10, 15 and 20 g/kg MA extract (**A**–**E**) for eight weeks without *V. cholerae* challenge, and of individuals challenged with the same pathogen under the same dietary treatments (**F**–**J**) for seven days, respectively. Staining: Hematoxylin-Eusine (H&E). Scale bar: 25 µm. Magnification: 10×. Abbreviations—MMCsA: Melanomacrophage center area, MMCsD: Melanom-acrophage center diameter, Ie: Interstitial edema, NMs: Necrotic macrophages, PSe: Perivascular spaces with edema, FMsn: Focal macrophage necrosis, Ce: Splenic capsule.

Figure 6 shows splenic sections before and after the challenge with *V. cholerae*. Before the challenge, the control (0 g/kg) displayed preserved architecture with an intact capsule, no necrosis or oedema, and MMCs of low size and density. In the supplemented groups (5–20 g/kg), a progressive increase in MMCs density/size was observed, together with improved organisation of sinusoids and trabeculae, without evident degenerative lesions.

After the challenge, the control exhibited acute alterations: interstitial oedema, diffuse necrosis, vascular dilatation, disorganisation and reduction of MMCs, together with erythrocyte infiltration. In the supplemented groups, a dose-dependent preservation gradient was evident: at 5 g/kg, limited foci of necrosis/oedema persisted; at 10–15 g/kg, lesions were markedly reduced, with a preserved capsule and organised MMCs; at 20 g/kg, the architecture remained practically intact, with minimal lesions and increased, well-defined MMCs. The micrographs show better structural preservation of the spleen and greater organisation of MMCs in the supplemented groups compared with the post-challenge control, in agreement with the morphometric findings.

Table 11 presents the morphometric variables of the MMCs before and after the challenge with *V. cholerae*. Before the challenge, significant differences among treatments were observed for AMMCs, AMLC, PR, and the LD and SD diameters (*p* < 0.05), with higher values at 15–20 g/kg than in the control. The SF did not differ among groups (*p* > 0.05). After the challenge, significant differences persisted for AMLC, PR, LD and SD (*p* < 0.05). The 15–20 g/kg treatments recorded the highest values for MMCs size and extent, surpassing the control. SF remained without differences (*p* > 0.05), indicating broadly similar morphology among treatments.

### 3.11. Multivariate Patterns of Functional Response

**Figure 7:**
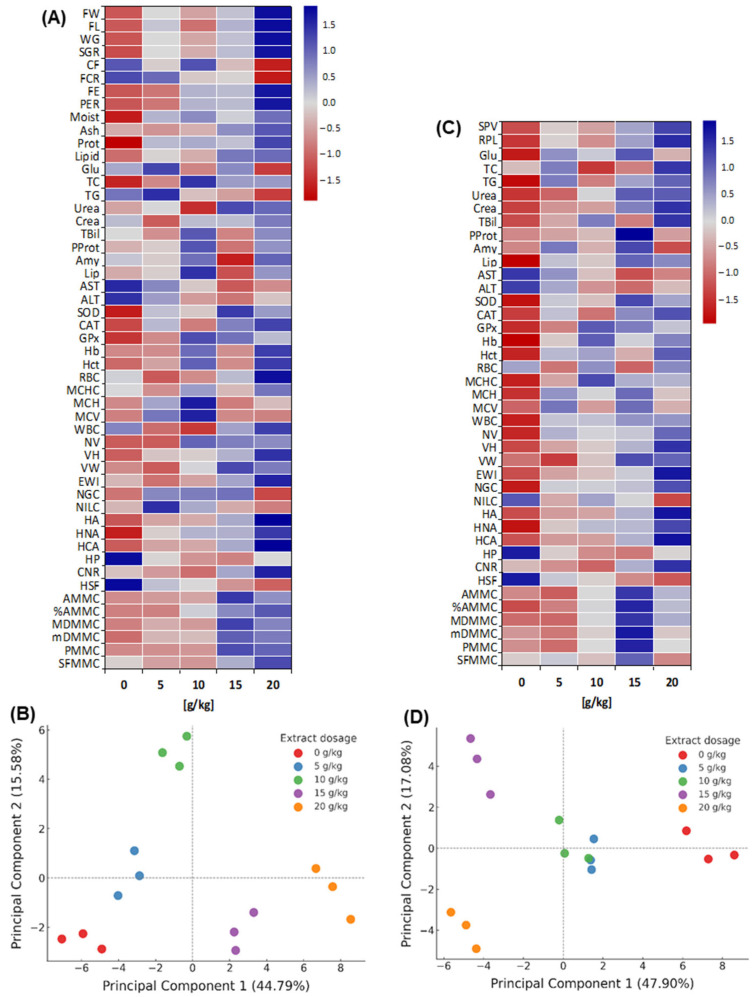
Multivariate patterns (Heatmaps and PCA) of *D. latifrons* fed diets supplemented with MA extract before and after *V. cholerae* challenge ((**A**,**B**): before challenge; (**C**,**D**): after challenge). Abbreviatures: FW: Final Weight; FL: Final Length; WG: Weight Gain; SGR: Specific Growth Rate; CF: Condition Factor; FCR: Feed Conversion Ratio; FE: Feed Efficiency; PER: Protein Efficiency Ratio; Moist: Moisture; Ash: Ash; Prot: Protein; Lipid: Lipid; Glu: Glucose; TC: Total Cholesterol; TG: Triglycerides; Urea: Urea; Crea: Creatinine; TBil: Total Bilirubin; Amy: Amylase; Lip: Lipase; AST: Aspartate Aminotransferase; ALT: Alanine Aminotransferase; SOD: Superoxide Dismutase; CAT: Catalase; GPx: Glutathione Peroxidase; Hb: Hemoglobin; Hct: Hematocrit; RBC: Erythrocytes; MCHC: Mean Corpuscular Hemoglobin Concentration; MCH: Mean Corpuscular Hemoglobin; MCV: Mean Corpuscular Volume; WBC: Total Leukocytes; NV: Number of Intestinal Villi; VH: Villus Height; VW: Villus Width; EWI: Enterocyte Width; NGC: Number of Goblet Cells, NILC: Number of Intraepithelial Lymphocyte Cells; HA: Hepatocyte Area; HNA: Hepatocyte Nucleus Area; HCA: Hepatocyte Cytoplasmic Area; HP: Hepatocyte Perimeter; CNR: Cytoplasmic-to-Nuclear Area Ratio; HSF: Hepatocyte Shape Factor; NMMC: Number of Melanomacrophage Centers; AMMC: Melanomacrophage Center Area; %AMMC: Percentage of Melanomacrophage Center Area; MDMMC: Major Diameter of Melanomacrophage Centers; mDMMC: Minor Diameter of Melanomacrophage Centers; PMMC: Perimeter of Melanomacrophage Centers; SFMMC: Shape Factor of Melanomacrophage Centers.

Before challenge (heatmap, Figure 7A); multivariate differences with a dose-dependent gradient were observed (*p* < 0.05): 0–10 g/kg with predominantly negative values and 15–20 g/kg positive. During growth, FW increased from −1.08 to +1.67, FL from −1.06 to +1.70 and SGR from −1.15 to +1.63; FCR decreased (minimum −1.53 at 20 g/kg) in parallel with increases in FE/PER (up to +1.60). In body composition, protein changed from −1.71 to +1.18 and lipids from −0.92 to +0.84. In enzymes, amylase/lipase were maximal at 10 g/kg, while AST/ALT went from positive to negative values at 15–20 g/kg. In antioxidants, SOD/CAT increased (−1.5/−1.2 to +1.2); in haematology, Hb/Hct increased from negative to +1.2; and in histomorphology, VH/HA increased (up to +1.35/+1.78). The pattern favored 15–20 g/kg.

Before the challenge (PCA, Figure 7B), the first two components explained > 60% of the variance (PC1 dominant). Distribution along PC1 separated 15–20 g/kg (positive extreme) from 0–5 g/kg (negative extreme), with 10 g/kg in an intermediate position. Positive PC1 loadings were associated with growth (FW, SGR), efficiencies (FE, PER) and histological traits; negative loadings with ALT/AST, bilirubin, urea, creatinine and lipids.

After the challenge (heatmap, Figure 7C), multivariate differences among treatments persisted (*p* < 0.05) with a dose-dependent pattern: the control showed predominantly negative values, whereas 10–15 g/kg shifted to positive, and 20 g/kg tended towards the mean for several variables. In biochemistry, AST/ALT were maximal in the control (Z = +1.44/+1.37) and declined to negative values at 15 g/kg; plasma proteins increased from −0.67 to +1.96; bilirubin and creatinine moved from −1.09/−1.17 to +1.47/+1.48; glucose and triglycerides peaked at +1.14 and +1.03 in 15–20 g/kg. SOD/CAT/GPx showed maxima of ~+1.2 at 10–15 g/kg. In haematology (Hb, Hct) and liver morphohistology (HA, HNA), Z-scores were positive (e.g., HA > +1.7), with lesion scores (e.g., necrosis) below −1.0. MMCs indices were highest at 15–20 g/kg.

The PCA after the challenge (Figure 7D) explained 47.90% (PC1) and 17.08% (PC2) of the variance (64.98% total). The control occupied the positive extreme of PC1, associated with damage/stress markers such as ALT/AST, bilirubin, urea and creatinine, whereas 5–10 g/kg lay centrally. By contrast, negative loadings grouped survival, plasma proteins, antioxidant, haematological and morphohistological traits. The model discriminated the treatments, with 15 g/kg positioned in the upper left (maximal separation from the control) and 20 g/kg in the lower left (close to 15 g/kg, differing on PC2).

The Z-score heatmap and PCA evidenced significant multivariate separation among treatments both before and after the challenge, with 15–20 g/kg concentrating the highest Z-scores for productive performance and tissue traits, and the lowest Z-scores for damage-indicative enzymes.

## 4. Discussion

### 4.1. Phytochemical Profile and Antibiogram Activity 

The phytochemical screening of the MA extract revealed a predominant presence of compounds widely recognised for their capacity to modulate microbial and cellular physiology. This profile aligns with previous reports in which extracts obtained using polar solvents (ethanol or methanol) showed a high concentration of total polyphenols especially morin, rutin, quercetin and chlorogenic acid molecules with strong antioxidant reactivity and the ability to disrupt bacterial membrane integrity [41,81]. In this context, the observed combination of secondary metabolites suggests a synergy of antimicrobial mechanisms that could explain the dose-dependent inhibition zones against *V. cholerae* obtained in this study.

Flavonoids and terpenes have been shown to exert bactericidal effects through depolarisation of the cytoplasmic membrane, chelation of essential metal ions, and inhibition of key enzymes involved in bacterial replication [50,55,82]. These compounds also interfere with the biosynthesis of fatty acids and proteins, leading to a progressive metabolic collapse in Gram-negative bacteria [83]. Consistent with this, the MA extract in our work exhibited an increasing inhibitory effect as concentration increased, evidencing a dose-dependent behaviour typical of extracts rich in flavonoids and tannins, as reported by Balouiri et al. [51] against *Vibrio parahaemolyticus* and *A. hydrophila* strains.

Moreover, the absence of inhibition in the negative control (ethanol) rules out the solvent as an active agent and confirms the intrinsic activity of the extract. This pattern is consistent with findings for MA extracts tested against pathogenic enteric bacteria such as *Escherichia coli*, *Salmonella typhi* and *V. cholerae*, where inhibition zones ranged between 10 and 15 mm at similar concentrations [29,56]. Likewise, the presence of alkaloids and quinones strengthens the hypothesis of a multifactorial bactericidal action, since these compounds possess redox capacity and can generate reactive oxygen species that compromise the bacterial cell wall [42,52]. Despite producing smaller inhibition zones than tetracycline, the use of MA extract in aquaculture may offer complementary advantages, such as a lower risk of selecting antibiotic-resistant strains due to its multicomponent, multitarget nature, the potential for synergistic interactions with conventional antimicrobials, and a presumably lower toxicity for fish, farm workers and the surrounding environment.

### 4.2. Modulation of Growth Kinetics, Virulence Markers and Cellular Morphology 

The results obtained demonstrate that the MA extract, at ½ MIC and MIC, inhibits the growth of *Vibrio cholerae* in a concentration-dependent manner and compromises cellular integrity. The combined evidence from growth kinetics and SEM micrographs indicates that this effect is exerted primarily at the level of the bacterial envelope, leading to loss of structural integrity and, ultimately, loss of viability. This inhibitory response is consistent with the nature of MA bioactive components, which have previously been described as modulators of membrane structure and permeability in pathogenic bacteria [21,35]. In this regard, plant extracts rich in phenolic compounds have been reported to increase *V. cholerae* envelope permeability, induce membrane hyperpolarisation and decrease cytoplasmic pH and intracellular ATP levels, thereby triggering an energetic collapse incompatible with sustained cell viability [19]. The marked inhibition of growth observed at the MIC, together with the severe morphological disruption documented by SEM, aligns well with such membrane-targeting and energy-depleting mechanisms.

Moreover, the repression of *toxR* and *ompU* observed in this study suggests that MA extract also alters the virulence regulatory systems of *V. cholerae*. *toxR* is a master regulator that controls the expression of multiple virulence factors, including *ompU*, which encodes an outer membrane porin essential for adaptation to host-associated conditions such as exposure to bile salts and other environmental stressors [67,84]. The concomitant decrease in *toxR* and *ompU* under treatment with MA extract is consistent with a scenario in which reduced *toxR* availability limits the transcriptional activation of *ompU* and other *toxR*-dependent virulence genes. Analogously, natural compounds such as capsaicin have been shown to modulate global virulence regulons in *V. cholerae*, decreasing the expression of toxigenic genes and modifying the activity of nucleoprotein regulators such as H-NS [85]. In line with this evidence, the present findings support the interpretation that MA extract not only affects bacterial survival, but also reshapes the regulatory hierarchy associated with virulence.

Furthermore, plant-derived polyphenols have been associated with the induction of pro-oxidant conditions in bacteria, either through auto-oxidation or through interactions with intracellular redox systems, thereby promoting the generation of reactive oxygen species (ROS) capable of damaging lipids, proteins and nucleic acids [21]. In *V. cholerae*, *ompU* has been implicated in tolerance to host-derived stress and in survival under hostile environmental conditions [66,84]. In this context, the reduction in *ompU* expression observed at the MIC in the present work can be interpreted as a decrease in the bacterium’s capacity to cope with stress, including the potential accumulation of ROS triggered by exposure to MA extract. The pronounced morphological alterations observed by SEM at the MIC, characterised by collapsed cells, disrupted envelopes and abundant debris, are compatible with this notion of cumulative damage to the cell surface and underlying structures.

Beyond direct antimicrobial and antivirulence mechanisms, interference with quorum sensing (QS)-regulated pathways also emerges as a plausible complementary mode of action in light of previous reports on phenolic compounds. Several plant-derived phenolics have been shown to disrupt QS-regulated processes in vibrios and other Gram-negative bacteria, reducing biofilm formation and repressing virulence factor production without necessarily exerting a strictly bactericidal effect. For *Morus* spp., compounds such as moracins have been linked to the modulation of AI-2-type communication circuits in vibrios [86], while extracts from *Mentha piperita* and *Myrtus communis* have been reported to inhibit acyl-homoserine lactone–mediated QS pathways in *Pseudomonas aeruginosa*, attenuating the expression of QS-regulated traits and biofilm formation [87,88]. Although specific QS markers and autoinducer levels were not assessed in the present study, the coordinated repression of *toxR* and *ompU*, together with the alterations in growth dynamics and cell morphology, is compatible with the possibility that MA extract also interacts with regulatory networks sensitive to cell-density cues and stress-related signalling.

### 4.3. Growth Outcomes and Protection Response

The gains in growth and feed efficiency at higher MA levels likely reflect improved digestive capacity and metabolic economy, in line with reports in *Oreochromis niloticus*, *Carassius carassius* and *Carassius auratus*, where mulberry extracts or meals enhanced growth performance [33,47,89]. These anabolic effects can be attributed to the presence of flavonoids, triterpenes and phenolic compounds that improve nutrient digestibility and stimulate intestinal and pancreatic enzyme secretion, as reported by Fu et al. [32] and Tingsen et al. [89] in *Micropterus salmoides* supplemented with mulberry extract.

Concomitantly, SGR and PER increased significantly at the higher doses, whereas FCR decreased. These results suggest improved protein and lipid metabolism, possibly mediated by positive regulation of transaminases and digestive enzymes, whose pre-challenge increases support greater digestive and metabolic capacity. This translates into an optimised energy balance and nutrient utilisation, consistent with Neamat-Allah et al. [33]. The findings for survival and RLP following challenge with *V. cholerae* confirm the immunoprotective role of the plant extract and its potential as a prophylactic agent against bacterial infections. Previous studies have shown that MA secondary metabolites can modulate innate immunity by increasing phagocytosis, lysozyme activity and controlled production of reactive oxygen species, thereby reducing bacterial colonisation [46,90].

These effects contribute to induced physiological resistance mechanisms, possibly associated with preservation of epithelial integrity and reduction of oxidative stress, as corroborated by the histological sections in the present study. In other species, such as *Cyprinus carpio* and *Clarias gariepinus*, supplementation with flavonoid-rich extracts has improved post-challenge survival by reducing bacterial load and maintaining immunometabolic homeostasis [34,45].

### 4.4. Proximate Composition of the Eviscerated Body

The patterns observed in proximate composition in *D. latifrons* suggest improved metabolic efficiency and nutrient utilisation. The increase in body protein at 15 and 20 g/kg agrees with Tingsen et al. [61], who reported that MA extract supplementation increased muscle protein and reduced ash content, indicating a better nitrogen balance. This effect may be attributed to the antioxidant action of polyphenols, which reduce lipid peroxidation and preserve cell membrane integrity, preventing oxidative proteolysis [48]. In *O. niloticus*, Neamat-Allah et al. [33], likewise documented an increase in body protein associated with improved nutrient bioavailability and hepatic stability derived from the extract’s hepatoprotective action.

Several studies have reported that MA secondary metabolites can stimulate not only the expression of digestive enzymes but also amino acid transporters in the intestinal epithelium, thereby promoting muscle protein synthesis and regulated lipid deposition [41,43]. Accordingly, the moderate increase in lipids in the supplemented groups may reflect a state of metabolic homeostasis rather than excessive fat accumulation, given that MA modulates hepatic lipogenesis by inhibiting key enzymes such as acetyl-CoA carboxylase (ACC) and hepatic lipase (LIP), while stimulating mitochondrial β-oxidation [48]. This functional duality optimises energy metabolism and protein utilisation efficiency, resulting in greater growth performance.

Likewise, the stability of ash content indicates that supplementation did not disrupt whole-body mineral balance an important consideration, as certain plant extracts may induce chelation or interfere with trace element absorption [91]. In this context, a balanced dietary formulation and moderate extract doses favoured conservation of tissue mineral status. Taken together, these patterns agree with previous reports in which inclusion of plant extracts rich in phenolic compounds improved body composition by increasing digestibility, reducing oxidative stress and enhancing nitrogen retention [48].

### 4.5. Plasma Biochemical Profile

The basal pattern showed reduced triglycerides and glucose at the highest inclusion levels (especially 15–20 g/kg), together with plasma protein levels higher than the control. This behaviour is consistent with the hypoglycaemic and hypolipidaemic activity attributed to compounds such as 1-deoxynojirimycin and polyphenols in MA, which inhibit α-glucosidases and, to a lesser extent, pancreatic lipase, thereby attenuating postprandial glucose entry and lipid absorption [47,92]. Multiple studies in fish and mammals have documented that mulberry leaf extracts activate AMPK-dependent energetic pathways that favour β-oxidation and fatty-acid utilisation while modulating lipogenesis [93,94], contributing to more controlled glycolysis, lower basal lipaemia and more efficient glucolipid homeostasis [41,94]. In this context, the fact that cholesterol and triglyceride profiles did not show a perfectly monotonic dose–response across all inclusion levels is compatible with metabolic homeostasis, where compensatory adjustments in synthesis, utilisation and clearance tend to buffer excessive changes in circulating lipids.

Similarly, the increase in plasma protein in supplemented fish is consistent with greater protein availability/functional hyperproteinaemia during the non-specific response, presumably via increased globulins and acute-phase proteins, as reported in tilapia supplemented with MA [33,46]. MA flavonoids (quercetin, rutin) may mitigate oxidative damage and sustain redox homeostasis, supporting humoral immunity and hepatic functionality [95,96]. Following the *V. cholerae* challenge, the observed variations in triglycerides, cholesterol, urea and creatinine reflect the interaction between infectious stress and the physiological response modulated by the plant extract. The increase in cholesterol and triglycerides in the extract groups may represent a compensatory mechanism of lipid synthesis for repairing cell membranes damaged during infection [97,98], while the intermediate values at moderate inclusion levels suggest a dynamic balance between lipid mobilisation and membrane restitution.

Nevertheless, plasma concentrations remained within physiological ranges, suggesting effective homeostatic control. The elevation of urea and creatinine at higher doses, without signs of toxicity, aligns with reports by Soor et al. [98], who observed in common carp an increase in these metabolites as an adaptive response to a higher rate of protein catabolism derived from immune activation. Regarding bilirubin, the modest between-treatment differences and slight post-challenge increase are compatible with subclinical choleretic/haemocatabolic changes typical of infectious processes, with no evidence of severe decompensation. These findings support the notion that MA optimises metabolism while sustaining the systemic response under infection [99].

### 4.6. Digestive and Metabolic, Enzymes and Antioxidant Activity

The enzymatic activities indicate a dual modulatory effect of MA: stimulation of digestive and antioxidant enzymes and attenuation of transaminases. Before the *V. cholerae* challenge, increases in amylase and lipase at 10–20 g/kg indicate greater digestive capacity and nutrient absorption, in line with findings in tilapia and carp fed MA extracts, where flavonoids and terpenes increased pancreatic activity and the efficiency of starch and lipid digestion [90].

Prior to the challenge, SOD, CAT and GPx rose at intermediate–high doses, indicating reinforcement of the antioxidant axis, consistent with activation of the Nrf2/Keap1 pathway that regulates antioxidant and glutathione-metabolism genes [89]. These findings agree with reports in *Carassius auratus* and *O. niloticus*, where flavonoid-rich extracts elevated SOD and GPx expression and reduced ROS and malondialdehyde [48,95]. CAT also showed a sustained increase, consistent with the decomposition of H_2_O_2_ produced by superoxide dismutation [75]. Following the *V. cholerae* challenge, amylase and lipase remained elevated, suggesting resilience of the exocrine–mucosal apparatus and energetic support to effector tissues. The post-challenge potentiation of SOD–CAT–GPx suggests containment of ROS associated with the inflammatory response; the literature on mulberry extracts reports higher antioxidant activity and lower peroxidation under stress/infection, frequently mediated by Nrf2 and attenuation of NF-κB [89,95,100]. Synergy among polyphenols, flavonoids and triterpenes could enhance NADPH-dependent enzymes and sustain intracellular redox homeostasis [45].

With respect to hepatic transaminases, the basal pattern, with slightly higher AST and ALT at intermediate inclusion levels and the lowest values at 20 g/kg, indicates that MA supplementation did not induce overt hepatocellular damage before the challenge and may even have attenuated subclinical leakage at the highest dose. After *V. cholerae* exposure, AST and ALT increased in all treatments, reflecting infection-associated hepatic stress; however, the highest activities in the control and the attenuated rise in fish receiving 15–20 g/kg point to a relative hepatoprotective effect of the extract under challenge [101,102]. In fish, increases in AST/ALT are interpreted as enzyme leakage secondary to hepatocellular damage; therefore, the lower post-challenge activities in MA-supplemented groups, together with the preservation of liver architecture described in the histological analysis, support better maintenance of tissue integrity under infectious stress. Importantly, AST and ALT values remained within physiological ranges, suggesting that MA mitigated, rather than prevented, infection-related liver damage. In tilapia supplemented with MA, declines in AST/ALT have been linked to polyphenols that stabilise membranes, limit lipid peroxidation and improve detoxification [33], which is coherent with the pattern observed here.

### 4.7. Haemato-Immunology

Before the challenge, the significant increases in HCT, Hb and RBC at 10 to 20 g/kg indicate greater oxygen-transport capacity and enhanced aerobic metabolism. This effect could derive from increased bioavailability of iron and copper, minerals essential for haemoglobin synthesis and the activity of metal-dependent redox enzymes [45]. Flavonoids present in MA have been shown to stimulate erythropoiesis by reducing oxidative damage in erythrocytes and preserving cell-membrane stability, thereby prolonging circulatory half-life [46,48].

Improvements in erythrocyte indices (MCV, MCH and MCHC) reinforce the interpretation of active, efficient haematopoiesis. These results agree with Sheikhlar et al. [45], who reported in *Clarias gariepinus* a significant increase in erythrocytes and haemoglobin following supplementation with MA leaves, suggesting activation of the erythropoietic axis mediated by stimulation of protein metabolism and iron balance. In this context, the maintenance of plasma osmolarity observed in the present study suggests that the doses used were safe and physiologically compatible with the fish’s haematological equilibrium.

Conversely, the leukocytosis detected in supplemented treatments reflects activation of the non-specific immune system, characterised by increased circulating lymphocytes, monocytes and neutrophils. Activation of these cells constitutes a primary response to bacterial infections, participating in phagocytosis, reactive oxygen species production and the release of pro-inflammatory cytokines [16]. Indeed, in vivo studies have shown that MA extracts increase gene expression of interleukin-1β (IL-1β), interferon-γ (IFN-γ) and tumour necrosis factor-α (TNF-α), enhancing cellular and humoral immunity [18,46].

After the *V. cholerae* challenge, fish fed the highest extract doses showed a smaller reduction in haematocrit and haemoglobin, alongside a sustained increase in WBC, confirming an effective immunoprotective response. This pattern is consistent with Miao et al. [99], who observed in *Oreochromis niloticus* increased resistance to *Streptococcus* agalactiae following dietary inclusion of mulberry leaf meal. Likewise, preservation of erythrocyte integrity in our work suggests reduced exposure to free radicals and oxidative haemolysis, in agreement with our SOD and CAT values [47]. The combined haematological and antioxidant response demonstrates that MA acts as a phytoimmunostimulant capable of reinforcing primary and secondary defence lines in fish. This effect may arise from interactions of antioxidant metabolites with redox-dependent signalling pathways (Nrf2, NF-κB), which modulate both cytokine production and the proliferation of immune cells [103].

### 4.8. Intestinal Structural Integrity

The intestinal histoarchitecture of *D. latifrons* showed that dietary supplementation with MA extract favoured structural integrity and functional competence of the intestinal epithelium both before and after the *V. cholerae* challenge. This protective effect is reflected in the preservation of villi, the increase in goblet-cell numbers and the reduction of inflammatory or desquamative lesions, suggesting a trophic and anti-inflammatory mechanism mediated by phenolic metabolites. In fish, the intestinal mucosa constitutes the first immunophysiological barrier against enteric pathogens; therefore, its preservation under infectious stress is a direct indicator of intestinal well-being [104].

The greater abundance of goblet cells and the strong PAS-positive reaction observed at 15 and 20 g/kg indicate increased secretion of neutral mucins and glycoproteins essential components of the mucous layer that hinder bacterial adhesion and modulate the intestinal microbiota [105,106]. Flavonoids and triterpenes present in mulberry have been shown to stimulate mucin synthesis and the expression of genes associated with the epithelial barrier (muc2, occludin and claudin-3), strengthening intercellular junctions and reducing bacterial translocation [90,105]. Similarly, in tilapia fed polyphenol-rich extracts, increases in villus height and density have been described, associated with improved nutrient absorption and growth indices [46].

Maintenance of intestinal morphology after the bacterial challenge confirms that the extract also acts as a cytoprotective agent. MA polyphenols, particularly morin and chlorogenic acid are capable of modulating the intestinal inflammatory response by inhibiting activation of the NF-κB transcription factor and reducing production of pro-inflammatory cytokines such as TNF-α and IL-1β [48]. This anti-inflammatory effect prevents epithelial degeneration and loss of microvilli, a phenomenon frequently induced by *Vibrio* spp. [47]. The observed mucosal integrity aligns with the metabolic and enzymatic results obtained, the higher amylase and lipase activity in the supplemented groups is likely attributable to a greater absorptive surface and regulation of digestive enzyme secretion, as described in other teleosts fed phytobiotics [107].

### 4.9. Hepatic Structural Integrity

The hepatic histo-morphological patterns show that MA supplementation confers a basal advantage prior to challenge: hepatocytes with broad, homogeneous cytoplasm, preserved trabecular organisation, adequate sinusoidal permeability and consistent PAS positivity features compatible with greater glycogen reserves and a stable anabolic state. Morphometrically, intermediate high doses increased hepatocyte area and diameter, cytoplasmic area and the C:N ratio, together with a larger PAS-positive area fraction and preserved sinusoids; these traits are consistent with energetic efficiency, favourable glucolipid regulation and reinforcement of redox homeostasis attributed to MA [48,96]. In fish, MA polyphenols activate cytoprotective pathways (Nrf2/ARE) and attenuate NF-κB-associated inflammatory signalling [108], which explains the preserved microarchitecture observed in the supplemented groups.

After seven days post-infection with *V. cholerae*, controls and non-supplemented fish exhibited vacuolar degeneration, focal necrosis and loss of the cord-like pattern, with reduced cytoplasmic area, decreased PAS-positive fraction, relatively increased nuclear area (low C:N), collapsed sinusoids and diminished cord thickness lesions typical of oxidative-inflammatory stress in acute infections [109]. By contrast, 15–20 g/kg MA preserved the parenchyma: hepatocyte area/diameter and C:N ratio were maintained, the PAS-positive fraction remained high, and cords retained stable thickness, indicating maintenance of energy metabolism. MA flavonoids (morin, rutin, quercetin) and triterpenes inhibit lipid peroxidation and increase antioxidant enzymes [48], translating into less vacuolisation and leucocytic infiltration and better preservation of sinusoids and endothelium. Consistently, MA modulates Nrf2/Keap1 and reduces NF-κB activation [47], thereby attenuating inflammation and tissue damage.

Taken together, morphometric preservation (hepatocyte area/diameter, C:N ratio, PAS-positive fraction and cord thickness) and histological integrity favour hepatic synthesis of plasma proteins and complement components, linking histo-morphological integrity to biochemical functionality. This is congruent with the stability of plasma proteins and the reduction in transaminases observed, confirming the multifunctional hepatoprotective role of MA under basal and post-infection conditions [95,100].

### 4.10. Splenic Structural Integrity

In the supplemented treatments especially at 15 and 20 g/kg an organised splenic architecture was observed, with clear demarcation of red and white pulp, abundant melanomacrophages and active lymphoid tissue. This pattern suggests controlled immunostimulation and enhanced clearance of cellular debris, in contrast to the congestion and parenchymal disorganisation evident in post-infection control groups. MMCs typical teleost structures acting as sites of phagocytosis and storage of pigments derived from haemoglobin and lipid oxidation were more abundant and compact in supplemented fish, indicating a dynamic immune state and efficient phagocytic response [109].

The splenic stimulation induced by the extract may be attributed to mulberry polyphenolic and terpenoid compounds, which act on NF-κB and Nrf2 signalling pathways, modulating cytokine production and promoting differentiation of lymphocytes [110]. In Nile tilapia supplemented with polyphenol-rich plant additives (quinoa seeds/prickly pear peel), Ahmed et al. [111], documented improved disease resistance to Aeromonas sobria, with histological restoration of splenic tissue, activation/increased presence of MMCs around vessels, and reduced necrosis in supplemented groups—results comparable to those observed in the present study.

The observed splenic integrity aligns with the haematological and immunological values discussed above, particularly the increase in total leucocytes and plasma proteins. These indicators suggest that MA extract strengthened haematopoiesis and splenic phagocytic capacity, reducing *V. cholerae*-induced oxidative damage. Phenolic extracts from *Morus* spp. have been shown to enhance macrophage activity, expression of pattern-recognition receptors (PRRs) and release of microbicidal enzymes, reinforcing innate immunity [46,111]. Maintenance of a compact splenic histoarchitecture with abundant lymphocytes in the white pulp demonstrates that MA supplementation acts not only as an immunostimulant but also as an immunoprotectant, preventing lymphoid degeneration and fibrosis commonly observed under severe bacterial infections.

### 4.11. Multivariate Structure of Functional Response 

The multivariate analysis coherently integrated the digestive, metabolic, haematological, and antioxidant responses induced by *Morus alba* in *Dormitator latifrons*.

Z-score plots and PCA showed a clear separation between control and supplemented groups, both before and after the challenge. Pre-challenge, the 10–20 g/kg doses clustered with higher amylase, lipase, plasma proteins, and antioxidant activity (SOD, CAT, GPx), whereas the control and lower doses aligned with reduced values. This pattern indicates an optimised physiological state with efficient metabolism and stable redox homeostasis, consistent with AMPK/Nrf2 pathway modulation by *Morus* compounds [91,94].

Following the *V. cholerae* challenge, the proximity of supplemented groups in factorial space reflected resilience and stability to infectious stress, while the control displayed greater dispersion (dysregulation and systemic inflammation). The tight correlation among antioxidant (CAT, GPx), haematological (WBC, Hb), and histological variables (intestinal/hepatic integrity) supports an integrated response model wherein the extract coordinates redox defence and innate immunity to limit tissue damage. Moreover, the positive association of these axes with growth and survival indicates that performance benefits arise from oxidative-stress control and immunometabolic stability, in line with reports in teleosts fed *Morus* extracts [33,42]. Taken together, the multivariate model positions *M. alba* as a phytobiotic with nutraceutical value to strengthen health and performance in sustainable aquaculture.

## 5. Conclusions

MA extract exerted direct effects on *Vibrio cholerae*, as in vitro assays revealed alterations in bacterial growth, attenuation of virulence-associated gene expression and marked morphological damage in planktonic cells, indicating a clear impact on pathogen physiology and cell integrity. At the host level, dietary supplementation with MA extract induced a coordinated improvement in the metabolic, immunological and histophysiological responses of *D. latifrons* under challenge with *V. cholerae*. Integrated indicators showed that moderate-to-high inclusion levels (15–20 g/kg) optimised digestive efficiency, oxidative balance and systemic immunity, resulting in greater growth and survival without adverse metabolic effects. Multivariate patterns confirmed consistent convergence among digestive, metabolic and immune variables in the supplemented groups, supporting the classification of MA as a multifunctional phytobiotic capable of modulating host physiology via regulation of redox and immune pathways. Taken together, these host–pathogen findings position MA extract as a promising nutraceutical additive for tropical aquaculture, offering a sustainable strategy to strengthen fish health and resilience against *V. cholerae* while contributing to reduced reliance on conventional antimicrobials.

## Figures and Tables

**Table 1:** Primers used for the amplification of regulatory, virulence effector and housekeeping genes in *V. cholerae*.

Gene	Function	Locus_Tag	Primer Sequence (5′–3′)	Product Size (bp)	Reference
*toxR*	Virulence regulator	VC_RS01330	F: CGGAACCGTTTTGACGTATT R: CTCGCAATGATTTGCATGAC	139	[66]
*ompU*	Outer membrane porin	VC_RS00090	F: ACACCGTATAGGCTGTCATTG R: GTGCTGAAGCTCGCCTATCTC	125	[67]
*recA*	Housekeeping	VC_RS03260	F: ATTGAAGGCGAAATGGGCGATAG R: TACACATACAGTTGGATTGCTTGAGG	115	[68]

Note: Gene identification was performed using the RefSeq annotation of the *V. cholerae* O1 El Tor N16961 genome (NC_002505.1), employing the official locus_tags for each target.

**Table 2:** Qualitative phytochemical screening of MA extract.

Metabolite	Test/Reaction	Result
Anthraquinones (quinones)	Bornträger reaction	++
Total phenolics	Ferric chloride (FeCl_3_)	+
Coumarins	UV fluorescence (365 nm) after NH_3_ vapour exposure	–
Tannins	Gelatine/NaCl precipitation	+
Reducing sugars	Fehling’s test	+
Triterpenes and steroids	Liebermann–Burchard	+++
Free amino acids	Ninhydrin	+
Flavonoids	Shinoda	+++
Oils and fats	Sudan	–
Alkaloids	Dragendorff	+
	Mayer	–
	Wagner	+++

Analyses were performed in triplicate (*n* = 3). (–) absent; (+) low presence; (++) moderate presence; (+++) high presence.

**Table 3:** In vitro inhibitory effect of MA extract against *V. cholerae* evaluated by the disk diffusion method.

Antibiotic/Extract	Concentration (mg/mL)	Inhibition Zone Diameter (mm)
Tetracycline (30 µg)	-	18.30 ± 0.36
Extract of MA	12.50	7.12 ± 0.13
	25.00	8.71 ± 0.24
	50.00	8.75 ± 0.16
	100.00	13.93 ± 0.29
Ethanol (10 µg)	-	-

Results are reported as measures ± standard error of 4 groups per treatment (*n* = 4).

**Table 4:** Growth performance in *D. latifrons* fed diets supplemented with MA extract for 8 weeks.

Parameters	Dietary Extract Levels of MA, g/kg					*p*
	0	5	10	15	20	
Final Weight (g)	22.86 ± 0.29 ^a^	24.49 ± 0.10 ^b^	23.8 ± 0.27 ^b^	24.83 ± 0.06 ^bc^	27.57 ± 0.42 ^c^	0.0003
Final Height (mm)	115.51 ± 0.17 ^a^	124.42 ± 0.24 ^b^	116.8 ± 0.29 ^b^	125.68 ± 0.20 ^c^	137.71 ± 0.95 ^c^	0.0010
Weight Gain (g)	12.78 ± 0.28 ^a^	14.41 ± 0.09 ^b^	13.79 ± 0.26 ^b^	14.79 ± 0.08 ^bc^	17.52 ± 0.43 ^c^	0.0003
SGR (%/day)	1.46 ± 0.02 ^a^	1.58 ± 0.01 ^b^	1.55 ± 0.03 ^b^	1.62 ± 0.02 ^bc^	1.80 ± 0.03 ^c^	0.0004
CF	1.48 ± 0.03 ^a^	1.27 ± 0.02 ^a^	1.49 ± 0.07 ^b^	1.25 ± 0.03 ^b^	1.06 ± 0.03 ^c^	0.0002
FCR	1.49 ± 0.02 ^a^	1.47 ± 0.03 ^ab^	1.36 ± 0.03 ^bc^	1.36 ± 0.07 ^c^	1.24 ± 0.03 ^d^	0.0001
FE	0.67 ± 0.01 ^a^	0.68 ± 0.02 ^b^	0.74 ± 0.07 ^b^	0.73 ± 0.04 ^c^	0.81 ± 0.02 ^c^	0.0001
PER	1.92 ± 0.02 ^a^	1.95 ± 0.03 ^b^	2.11 ± 0.02 ^b^	2.10 ± 0.01 ^c^	2.31 ± 0.06 ^c^	0.0001

Results are reported as measures ± standard error of 3 groups per treatment (*n* = 3). Different letters (abcd) indicate significant differences (*p* < 0.05). Abbreviations: SGR: Specific growth rate, CF: Condition factor, FCR: Feed conversion ratio, FE: Feed efficiency ratio, PER: Protein efficiency ratio.

**Table 5:** Nutritional composition in *D. latifrons* fed diets supplemented with MA extract for 8 weeks.

Parameters	Dietary Extract Levels of MA, g/kg					*p*
	0	5	10	15	20	
Moisture (%)	73.5 ± 0.21 ^b^	75.59 ± 0.31 ^a^	76.04 ± 0.29 ^a^	75.31 ± 0.19 ^a^	76.36 ± 0.42 ^a^	0.0198
Ash (%)	1.41 ± 0.35 ^a^	1.22 ± 0.18 ^a^	1.44 ± 0.41 ^a^	2.43 ± 0.38 ^a^	2.93 ± 0.18 ^a^	0.1882
Protein (%)	16.24 ± 0.34 ^c^	19.89 ± 0.23 ^b^	20.13 ± 0.19 ^b^	19.75 ± 0.24 ^b^	21.89 ± 0.29 ^a^	0.0001
Lipid (%)	4.91 ± 0.05 ^c^	5.29 ± 0.23 ^c^	5.15 ± 0.05 ^bc^	5.77 ± 0.16 ^ab^	5.83 ± 0.20 ^a^	0.0322

Results are reported as measures ± standard error of 3 groups per treatment (*n* = 3). Different letters (abc) indicate significant differences (*p* < 0.05).

**Table 6:** Plasma biochemical parameters in *D. latifrons* fed diets supplemented with MA extract for 8 weeks and challenged for seven days with *V. cholerae*.

Parameters	Dietary Extract Levels of MA, g/kg					*p*
	0	5	10	15	20	
Before the challenge						
Cholesterol, mg/dL	143.5 ± 1.09 ^d^	162.83 ± 2.96 ^c^	218.01 ± 2.95 ^a^	192.83 ± 2.28 ^b^	193.5 ± 1.42 ^b^	0.0001
Triglycerides, mg/dL	289.5 ± 2.74 ^ab^	310.33 ± 2.84 ^a^	253.33 ± 0.54 ^bc^	245.67 ± 0.25 ^c^	222.33 ± 8.02 ^c^	0.0001
Glucose, mg/dL	36.67 ± 0.51 ^ab^	40.02 ± 0.33 ^a^	32.60 ± 0.84 ^b^	37.67 ± 0.51 ^ab^	30.07 ± 1.02 ^c^	0.0015
Bilirubin, mg/dL	0.07 ± 0.01 ^ab^	0.07 ± 0.01 ^ab^	0.09 ± 0.03 ^a^	0.06 ± 0.02 ^b^	0.08 ± 0.05 ^ab^	0.0001
Urea, mg/dL	4.33 ± 0.11 ^bc^	4.67 ± 0.11 ^abc^	3.67 ± 0.11 ^c^	5.67 ± 0.11 ^a^	5.44 ± 0.17 ^ab^	0.0004
Creatinine, mg/dL	0.21 ± 0.02 ^a^	0.17 ± 0.02 ^a^	0.23 ± 0.02 ^a^	0.23 ± 0.02 ^a^	0.27 ± 0.02 ^a^	0.3692
Proteins, g/dL	4.73 ± 0.09 ^a^	5.44 ± 0.04 ^b^	5.81 ± 0.11 ^b^	5.50 ± 0.03 ^b^	5.67 ± 0.1 ^b^	0.0025
After the challenge						
Cholesterol, mg/dL	123.33 ± 0.69 ^c^	134.83 ± 1.07 ^b^	113.17 ± 0.77 ^d^	118.67 ± 0.67 ^cd^	142.17 ± 0.67 ^a^	0.0001
Triglycerides, mg/dL	207.01 ± 1.09 ^d^	285.17 ± 3.91 ^ab^	234.05 ± 0.44 ^c^	273.33 ± 0.69 ^b^	292.67 ± 1.21 ^a^	0.0001
Glucose, mg/dL	51.67 ± 0.51 ^c^	58.02 ± 0.67 ^ab^	56.33 ± 0.51 ^b^	59.67 ± 0.51 ^a^	55.01 ± 0.67 ^b^	0.0021
Bilirubin, mg/dL	0.13 ± 0.02 ^b^	0.14 ± 0.01 ^b^	0.15 ± 0.09 ^ab^	0.13 ± 0.06 ^b^	0.16 ± 0.11 ^a^	0.0001
Urea, mg/dL	5.17 ± 0.04 ^c^	5.52 ± 0.08 ^c^	6.41 ± 0.04 ^b^	7.52 ± 0.03 ^a^	7.52 ± 0.14 ^a^	0.0001
Creatinine, mg/dL	0.11 ± 0.01 ^d^	0.18 ± 0.02 ^c^	0.19 ± 0.01 ^c^	0.34 ± 0.02 ^b^	0.43 ± 0.01 ^a^	0.0001
Proteins, g/dL	5.09 ± 0.02 ^c^	5.14 ± 0.02 ^c^	5.18 ± 0.02 ^bc^	5.74 ± 1.3 ^a^	6.13 ± 0.01 ^a^	0.0012

Results are reported as measures ± standard error of 3 groups per treatment (*n* = 3). Different letters (abcd) indicate significant differences (*p* < 0.05).

**Table 7:** Digestive, metabolic and antioxidant enzymes in *D. latifrons* fed diets supplemented with MA extract for 8 weeks and challenged for seven days with *V. cholerae*.

Parameters	Dietary Extract Levels of MA, g/kg					*p*
	0	5	10	15	20	
Before the challenge						
AST, U/L	35.02 ± 0.33 ^b^	39.00 ± 0.35 ^a^	40.87 ± 0.51 ^a^	39.67 ± 0.69 ^a^	31.33 ± 0.51 ^c^	0.0001
ALT, U/L	33.33 ± 0.51 ^b^	37.67 ± 0.19 ^a^	38.60 ± 0.50 ^a^	37.00 ± 0.33 ^a^	26.43 ± 1.57 ^c^	0.0001
AMY, U/L	33.07 ± 0.67 ^ab^	32.05 ± 0.67 ^b^	35.74 ± 0.72 ^ab^	27.56 ± 0.51 ^c^	36.00 ± 1.01 ^a^	0.0039
LIP, U/L	26.50 ± 0.24 ^bc^	27.33 ± 1.02 ^bc^	32.66 ± 0.60 ^a^	24.33 ± 0.69 ^c^	29.67 ± 0.84 ^ab^	0.0127
SOD, U/mL	128.96 ± 1.25 ^c^	139.99 ± 0.79 ^b^	137.55 ± 0.92 ^b^	148.3 ± 0.97 ^a^	142.32 ± 1.56 ^ab^	0.0031
CAT, U/mL	27.38 ± 1.02 ^c^	33.28 ± 0.61 ^b^	29.05 ± 0.73 ^bc^	35.44 ± 0.78 ^ab^	37.84 ± 0.95 ^a^	0.0101
GPx, U/mL	87.69 ± 1.61 ^c^	91.39 ± 0.78 ^c^	96.5 ± 0.65 ^b^	102.39 ± 0.86 ^a^	100.44 ± 0.88 ^a^	0.0220
After the challenge						
AST, U/L	82.50 ± 0.71 ^a^	80.50 ± 0.71 ^ab^	78.50 ± 0.24 ^bc^	76.67 ± 0.51 ^c^	77.50 ± 0.24 ^bc^	0.0383
ALT, U/L	80.33 ± 0.51 ^a^	78.01 ± 0.67 ^ab^	75.67 ± 0.51 ^b^	75.00 ± 0.67 ^b^	76.42 ± 0.51 ^b^	0.0233
AMY, U/L	85.21 ± 1.45 ^c^	95.23 ± 1.45 ^ab^	87.25 ± 0.33 ^bc^	98.02 ± 0.30 ^a^	82.33 ± 1.35 ^c^	0.0008
LIP, U/L	42.53 ± 0.81 ^d^	52.37 ± 0.84 ^bc^	50.24 ± 0.51 ^c^	57.41 ± 1.07 ^a^	55.67 ± 1.07 ^ab^	0.0004
SOD, U/mL	131.46 ± 1.35 ^c^	134.85 ± 1.68 ^c^	144.35 ± 1.25 ^b^	146.93 ± 1.55 ^b^	151.85 ± 1.71 ^a^	0.0050
CAT, U/mL	41.86 ± 0.83 ^c^	48.62 ± 0.64 ^b^	51.19 ± 0.61 ^b^	50.54 ± 0.93 ^b^	61.44 ± 0.79 ^a^	0.0155
GPx, U/mL	178.38 ± 1.22 ^d^	184.37 ± 1.13 ^c^	203.04 ± 1.39 ^a^	202.76 ± 1.44 ^ab^	198.96 ± 1.32 ^b^	0.0001

Results are reported as measures ± standard error of 3 groups per treatment (*n* = 3). Different letters (abcd) indicate significant differences (*p* < 0.05). Abbreviations: AST: Aspartate aminotransferase, ALT: Alanine aminotransferase, AMY: Amylase, LIP: Lipase, SOD: Superoxide Dismutase, CAT: Catalase, GPx: Glutathione Peroxidase.

**Table 8:** Haematology in *D. latifrons* fed diets supplemented with MA extract for 8 weeks and challenged for seven days with *V. cholerae*.

Parameters	Dietary Extract Levels of MA, g/kg					*p*
	0	5	10	15	20	
Before the challenge						
Haematocrit %	40.30 ± 0.27 ^b^	41.73 ± 0.56 ^b^	46.77 ± 0.44 ^a^	41.17 ± 0.85 ^b^	47.90 ± 0.49 ^a^	0.0006
Haemoglobin, g/dL	13.27 ± 0.34 ^b^	13.33 ± 0.35 ^b^	15.80 ± 0.23 ^a^	13.37 ± 0.37 ^b^	16.47 ± 0.32 ^a^	0.0043
RBC, ×10^12^/L	5.17 ± 0.05 ^bc^	4.60 ± 0.07 ^d^	4.80 ± 0.10 ^cd^	5.27 ± 0.10 ^b^	6.13 ± 0.05 ^a^	0.0001
MCV, fL	77.62 ± 0.12 ^c^	90.77 ± 1.03 ^b^	97.38 ± 2.02 ^a^	78.16 ± 0.36 ^c^	77.63 ± 0.22 ^c^	0.0004
MCH, pg/cell	25.66 ± 0.44 ^bc^	29.05 ± 1.01 ^b^	32.99 ± 0.76 ^a^	25.37 ± 0.43 ^c^	26.83 ± 0.30 ^bc^	0.0035
MCHC, g/dL	32.90 ± 0.64 ^a^	31.96 ± 0.79 ^a^	33.77 ± 0.18 ^a^	32.48 ± 0.69 ^a^	34.36 ± 0.35 ^a^	0.4787
WBC, ×10^9^/L	13.59 ± 0.12 ^bc^	13.12 ± 0.05 ^c^	12.77 ± 0.19 ^c^	14.34 ± 0.11 ^b^	15.17 ± 0.05 ^a^	0.0001
After the challenge						
Haematocrit %	30.63 ± 0.34 ^c^	32.37 ± 0.25 ^ab^	32.60 ± 0.15 ^ab^	31.70 ± 0.24 ^bc^	33.30 ± 0.19 ^a^	0.0105
Haemoglobin, g/dL	10.43 ± 0.12 ^d^	11.53 ± 0.11 ^c^	12.53 ± 0.11 ^a^	11.70 ± 0.07 ^bc^	12.27 ± 0.13 ^ab^	0.0001
RBC, ×10^12^/L	5.40 ± 0.12 ^a^	4.70 ± 0.09 ^a^	5.50 ± 0.15 ^a^	4.63 ± 0.25 ^a^	5.53 ± 0.16 ^a^	0.1103
MCV, fL	56.81 ± 0.12 ^c^	61.3 ± 0.09 ^ab^	65.31 ± 0.15 ^bc^	68.97 ± 0.25 ^a^	63.46 ± 0.16 ^abc^	0.0543
MCH, pg/cell	19.37 ± 0.43 ^b^	24.58 ± 0.40 ^a^	22.89 ± 0.66 ^ab^	25.67 ± 1.27 ^a^	22.25 ± 0.51 ^ab^	0.0434
MCHC, g/dL	34.08 ± 0.54 ^c^	35.63 ± 0.12 ^bc^	38.45 ± 0.33 ^a^	36.91 ± 0.09 ^ab^	36.83 ± 0.18 ^ab^	0.0019
WBC, ×10^9^/L	12.01 ± 0.43 ^c^	15.83 ± 0.15 ^abc^	15.9 ± 1.12 ^ab^	16.90 ± 0.75 ^a^	16.97 ± 0.56 ^a^	0.0046

Results are reported as measures ± standard error of 3 groups per treatment (*n* = 3). Different letters (abcd) indicate significant differences (*p* < 0.05). Abbreviations: RBC: Red Blood Cells, MCV: Mean Corpuscular Volume, MCH: Mean Corpuscular Hemoglobin, MCHC: Mean Corpuscular Hemoglobin Concentration. WBC: White Blood Cells.

**Table 9:** Histomorphology of the gut in *D. latifrons* fed diets supplemented with MA extract for 8 weeks and challenged for seven days with *V. cholerae*.

Parameters	Dietary Extract Levels of MA, g/kg					*p*
	0	5	10	15	20	
Before the challenge						
Number Villi, U	20.03 ± 0.05 ^b^	20.01 ± 0.33 ^b^	25.67 ± 1.02 ^a^	25.31 ± 0.33 ^ab^	24.67 ± 0.69 ^ab^	0.0044
Villus Height, µm	136.29 ± 3.43 ^b^	146.61 ± 2.9 ^b^	147.55 ± 3.99 ^b^	153.92 ± 3.11 ^ab^	169.77 ± 3.98 ^a^	0.0317
Villus Width, µm	43.41 ± 1.44 ^c^	40.54 ± 1.6 ^c^	48.76 ± 0.64 ^bc^	59.64 ± 2.81 ^a^	55.72 ± 2.62 ^ab^	0.0136
EWI, µm	11.47 ± 0.43 ^bc^	10.32 ± 0.57 ^bc^	11.14 ± 0.35 ^c^	13.16 ± 0.33 ^ab^	15.85 ± 0.73 ^a^	0.0084
NGC, /100 µm	8.14 ± 0.38 ^b^	10.16 ± 0.07 ^ab^	10.29 ± 0.47 ^ab^	10.44 ± 0.26 ^a^	7.67 ± 0.11 ^b^	0.0077
NILC, /100 µm	13.97 ± 0.48 ^c^	19.82 ± 0.67 ^a^	17.45 ± 0.69 ^ab^	15.8 ± 0.27 ^b^	15.11 ± 0.53 ^b^	0.0371
After the challenge						
Number Villi, U	19.05 ± 1.17 ^b^	23.67 ± 0.35 ^a^	22.83 ± 0.19 ^ab^	23.17 ± 0.63 ^a^	25.67 ± 0.92 ^a^	0.0413
Villus Height, µm	137.38 ± 3.38 ^c^	145.22 ± 2.18 ^bc^	148.37 ± 3.44 ^bc^	154.06 ± 0.5 ^b^	168.62 ± 0.2 ^a^	0.0036
Villus Width, µm	43.82 ± 0.75 ^bc^	41.05 ± 0.77 ^c^	47.87 ± 0.41 ^b^	58.53 ± 0.41 ^a^	57.00 ± 0.72 ^a^	0.0001
EWI, µm	13.44 ± 0.11 ^c^	14.29 ± 0.27 ^b^	14.58 ± 0.02 ^bc^	15.04 ± 0.11 ^b^	17.44 ± 0.19 ^a^	0.0001
NGC, /100 µm	16.78 ± 0.20 ^c^	19.84 ± 0.93 ^b^	19.82 ± 0.07 ^b^	20.07 ± 0.28 ^ab^	22.25 ± 0.18 ^a^	0.0074
NILC, /100 µm	26.34 ± 0.62 ^a^	21.46 ± 1.62 ^bc^	24.14 ± 0.39 ^ab^	22.74 ± 0.12 ^abc^	19.35 ± 0.35 ^c^	0.0412

Results are reported as measures ± standard error of 3 groups per treatment (*n* = 3). Different letters (abc) indicate significant differences (*p* < 0.05). Abbreviations: EWI: Enterocyte Width, NGC: Number of Goblet Cells, NILC: Number of Intraepithelial Lymphocyte Cells.

**Table 10:** Histomorphology of the liver in *D. latifrons* fed diets supplemented with MA extract for 8 weeks and challenged for seven days with *V. cholerae*.

Parameters	Dietary Extract Levels of MA, g/kg					*p*
	0	5	10	15	20	
Before the challenge						
HA, µm^2^	51.35 ± 0.34 ^b^	55.32 ± 1.72 ^b^	55.67 ± 2.51 ^b^	62.74 ± 1.08 ^b^	84.95 ± 5.03 ^a^	0.0031
HNA, µm^2^	5.92 ± 0.03 ^b^	5.32 ± 0.02 ^c^	5.97 ± 0.05 ^b^	6.34 ± 0.08 ^a^	6.47 ± 0.09 ^a^	0.0003
HCA, µm^2^	45.43 ± 0.37 ^b^	50.00 ± 1.74 ^b^	49.70 ± 2.55 ^b^	56.40 ± 1.09 ^b^	78.48 ± 4.97 ^a^	0.0035
HP, µm	25.99 ± 0.24 ^c^	28.32 ± 0.59 ^bc^	29.60 ± 0.94 ^bc^	31.02 ± 0.36 ^b^	35.96 ± 0.99 ^a^	0.0019
RCN, µm^2^	7.67 ± 0.09 ^b^	9.41 ± 0.37 ^b^	8.35 ± 0.49 ^b^	8.90 ± 0.23 ^b^	12.09 ± 0.66 ^a^	0.0138
After the challenge						
HA, µm^2^	46.34 ± 0.61 ^d^	53.20 ± 0.22 ^c^	53.87 ± 0.30 ^c^	63.16 ± 0.70 ^b^	81.41 ± 0.38 ^a^	0.0001
HNA, µm^2^	3.89 ± 0.06 ^c^	4.630 ± 0.13 ^b^	4.89 ± 0.11 ^b^	4.90 ± 0.10 ^b^	5.53 ± 0.12 ^a^	0.0012
HCA, µm^2^	42.45 ± 0.67 ^d^	48.56 ± 0.19 ^c^	48.98 ± 0.27 ^c^	58.26 ± 0.80 ^b^	75.88 ± 0.28 ^a^	0.0001
HP, µm	28.39 ± 0.69 ^a^	24.85 ± 0.16 ^b^	23.99 ± 0.19 ^b^	23.75 ± 0.04 ^b^	24.95 ± 0.25 ^b^	0.0018
RCN, µm^2^	10.96 ± 0.34 ^bc^	10.53 ± 0.31 ^bc^	10.05 ± 0.23 ^c^	11.93 ± 0.42 ^b^	13.75 ± 0.26 ^a^	0.0056

Results are reported as measures ± standard error of 3 groups per treatment (*n* = 3). Different letters (abcd) indicate significant differences (*p* < 0.05). Abbreviations: HA: Hepatocyte area, HNA: Hepatocyte nucleus area, HCA: Cytoplasm area, HP: Perimeter, RCN: Ratio of cytoplasm area to nucleus area of the hepatocyte.

**Table 11:** Histomorphology of the spleen in *D. latifrons* fed diets supplemented with MA extract for 8 weeks and challenged for seven days with *V. cholerae*.

Parameters	Dietary Extract Levels of MA, g/kg					*p*
	0	5	10	15	20	
Before the challenge						
AMMCs, µm^2^	800.95 ± 10.98 ^b^	847.63 ± 10.92 ^b^	879.44 ± 10.35 ^b^	1710.65 ± 11.69 ^a^	1351.13 ± 13.53 ^ab^	0.0350
AMLC, %	9.07 ± 2.18 ^b^	9.11 ± 0.72 ^b^	12.64 ± 0.93 ^ab^	15.55 ± 0.92 ^ab^	17.61 ± 1.46 ^b^	0.0950
LD, µm	31.05 ± 4.01 ^c^	34.74 ± 1.65 ^bc^	33.23 ± 1.06 ^bc^	53.75 ± 2.43 ^a^	47.05 ± 4.04 ^ab^	0.0373
SD, µm	21.62 ± 1.38 ^b^	24.62 ± 2.03 ^ab^	24.9 ± 1.18 ^ab^	32.4 ± 2.33 ^a^	31.01 ± 0.85 ^a^	0.1018
PR, µm	103.50 ± 6.48 ^b^	104.73 ± 5.27 ^b^	106.32 ± 5.47 ^b^	161.8 ± 8.42 ^a^	155.18 ± 9.72 ^a^	0.0135
SF	1.14 ± 0.11 ^ab^	1.06 ± 0.02 ^ab^	1.05 ± 0.02 ^b^	1.24 ± 0.09 ^ab^	1.43 ± 0.05 ^a^	0.2070
After the challenge						
AMMCs, µm^2^	1260.07 ± 8.04 ^bc^	1083.07 ± 9.79 ^c^	1592.28 ± 9.54 ^bc^	2451.99 ± 11.17 ^a^	1778.97 ± 20.46 ^ab^	0.0103
AMLC, %	13.56 ± 0.25 ^d^	15.3 ± 0.83 ^cd^	18.57 ± 0.15 ^bc^	26.21 ± 0.55 ^b^	19.6 ± 1.13 ^a^	0.0002
LD, µm	44.01 ± 1.32 ^c^	43.75 ± 2.35 ^c^	51.54 ± 1.24 ^bc^	65.35 ± 1.21 ^a^	55.02 ± 2.39 ^b^	0.0032
SD, µm	30.47 ± 1.14 ^b^	27.93 ± 0.98 ^b^	35.02 ± 0.8 ^b^	57.13 ± 3.86 ^a^	34.43 ± 1.89 ^b^	0.0013
PR, µm	138.26 ± 3.8 ^b^	130.49 ± 6.85 ^b^	154.98 ± 5.2 ^b^	209.78 ± 9.04 ^a^	154.92 ± 4.84 ^b^	0.0033
SF	1.22 ± 0.04 ^a^	1.26 ± 0.02 ^a^	1.21 ± 0.02 ^a^	1.43 ± 0.06 ^a^	1.14 ± 0.10 ^a^	0.3587

Results are reported as measures ± standard error of 3 groups per treatment (*n* = 3). Different letters (abcd) indicate significant differences (*p* < 0.05). Abbreviations: AMMCs: Area of melanomacrophage centers, AMLC: % Area of melanomacrophage centers, LD: Largest diameter, SD: Smallest diameter, PR: Perimeter, SF: Shape factor.

## Data Availability

The data supporting the findings are contained within the article. Additional datasets may be requested from the corresponding author upon reasonable request.
